# Comprehensive and scalable quantification of splicing differences with MntJULiP

**DOI:** 10.1186/s13059-022-02767-y

**Published:** 2022-09-14

**Authors:** Guangyu Yang, Sarven Sabunciyan, Liliana Florea

**Affiliations:** 1 Department of Computer Science, Johns Hopkins University, 733 N Broadway, MRB 462, Baltimore, MD 21205 USA; 2 Department of Pediatrics, Johns Hopkins School of Medicine, 600 N Wolfe St, Blalock 1147, Baltimore, MD 21287 USA; 3 McKusick-Nathans Department of Genetic Medicine, Johns Hopkins School of Medicine, 733 N Broadway, MRB 453, Baltimore, MD 21205 USA; 4 Department of Computer Science, Johns Hopkins University, Baltimore, MD 21205 USA

**Keywords:** Alternative splicing, Differential splicing, Transcriptomics, RNA-seq

## Abstract

Tools for differential splicing detection have failed to provide a comprehensive and consistent view of splicing variation. We present MntJULiP, a novel method for comprehensive and accurate quantification of splicing differences between two or more conditions. MntJULiP detects both changes in intron splicing ratios and changes in absolute splicing levels with high accuracy, and can find classes of variation overlooked by other tools. MntJULiP identifies over 29,000 differentially spliced introns in 1398 GTEx brain samples, including 11,242 novel introns discovered in this dataset. Highly scalable, MntJULiP can process thousands of samples within hours to reveal splicing constituents of phenotypic differentiation.

**Supplementary Information:**

The online version contains supplementary material available at 10.1186/s13059-022-02767-y.

## Background

Gene alternative splicing is a fundamental biological process that gives rise to a wide array of protein isoforms with modified properties in plant and animal systems. More than 95% of human genes are alternatively spliced, and high levels were reported in virtually all sequenced eukaryotic species. Most splicing variations are tissue specific, but splicing is also altered by external stimuli [1] and aberrant splicing has been associated with diseases [2]. Therefore, there is a great need to accurately map and quantify gene splice variants, as well as to identify differences in splicing between conditions.

Current methods aim to detect and quantify alternative splicing from RNA sequencing (RNA-seq) data at the level of transcripts (isoforms), splicing events (exon skipping, mutually exclusive exons, alternative exon ends, intron retention), or primitive features (subexons, introns). Isoform-level quantification methods (Cuffdiff, Cuffdiff2, MISO, Sleuth [3–6]) require a reference annotation or a reconstructed set of transcripts, and their performance suffers from incompleteness and inaccuracies in the assemblies. Event level methods (DiffSplice, rMATS, SUPPA2 [7–9]) are less affected by assembly errors, but represent only a subset of alternative splicing variations. For both of these classes of methods, quantification is further complicated by the ambiguity in assigning reads that map to multiple locations in the genome and multiple transcripts of a gene. In contrast, more recent methods (LeafCutter, MAJIQ, JunctionSeq [10–12]) target introns, which can be more reliably identified from read alignments, capture a wider variety of splicing variations, and are less ambiguous to quantify, as intron-spanning reads associate with unique splice patterns. Methods further differ in how they define splicing differences. Most methods quantify changes in the relative splicing levels of the target feature within a group of mutually exclusive local splicing patterns (LeafCutter, MAJIQ, rMATS, SUPPA2, DiffSplice), or identify features whose splicing levels are inconsistent with the rest of the gene (JunctionSeq, DEXseq [13]), all of which are reflected as differences in splicing ratios. Yet others look for changes in the overall (absolute) abundance levels of a feature [3, 4, 6, 14], to identify isoforms whose changes in abundance lead to functional effects, known as isoform-level regulation. Lastly, to increase accuracy, some methods rely on a pre-existing set of gene annotations to identify relevant splicing variations, which limits the discovery of novel and potentially condition-specific features. This rich spectrum of methods for alternative splicing quantification and differential analysis offers a diverse but often inconsistent view of alternative splicing variation [15].

We introduce MntJULiP, a statistical learning method based on a novel mixture Bayesian framework, for detecting differences in splicing between large collections of RNA-seq samples. MntJULiP represents splicing variation at the intron level, thus capturing most types of splicing variation while avoiding the pitfalls of assembly. It infers intron annotations directly from the alignments, making it possible to discover new unannotated candidate markers. MntJULiP detects both differences in intron abundance levels, herein called *differential splicing abundance* (DSA), and differences in intron splicing ratios relative to the local gene output, termed *differential splicing ratio* (DSR). Salient features of MntJULiP include:

(i) A novel statistical framework, including a zero-inflated negative binomial mixture model for individual introns, in the DSA model, and a Dirichlet multinomial mixture model for groups of alternatively spliced introns, in the DSR model;
(ii) It captures significantly more alternative splicing variation, and more types of variation, than existing tools;
(iii) Superior performance compared to reference methods, including increased sensitivity in control experiments, and high reproducibility and reduced false positives in comparison with real data;
(iv) A unique mixture model that allows comparison of multiple conditions simultaneously, to aptly capture global variation in complex and time-series experiments; and
(v) Highly scalable, it processed hundreds of GTEx samples in less than half an hour, and the full set of 1398 GTEx samples in less than a day.

We assess the performance of MntJULiP and several reference programs on simulated and real RNA-seq data, with varying degrees of splice variation and different dataset sizes. We include in the comparisons, as feasible, the state-of-the-art intron-based tools LeafCutter, MAJIQ, and the event-based rMATS and SUPPA2, and Cuffdiff2 and Sleuth as tools compatible with the DSA test. We illustrate MntJULiP’s ability to detect more types of alternative splicing variation in the comparison of hippocampus samples from healthy and epileptic mice. We then demonstrate MntJULiP’s capability for simultaneous multi-condition comparisons in a 7-point time-series experiment on differentiating mouse taste organoids, and its ability to handle large data sets on a collection of RNA-seq samples from four human tissues obtained from the GTEx project. Lastly, to demonstrate its power and usefulness, we apply MntJULiP to the 1398 GTEx RNA-seq samples from 13 brain regions to characterize the landscape of alternative splicing variation in this tissue.

MntJULiP is implemented in Python and is distributed free of charge under a GPL license from https://github.com/splicebox/MntJulip/.

## Results

### Performance evaluation on simulated data

**Fig. 1:**
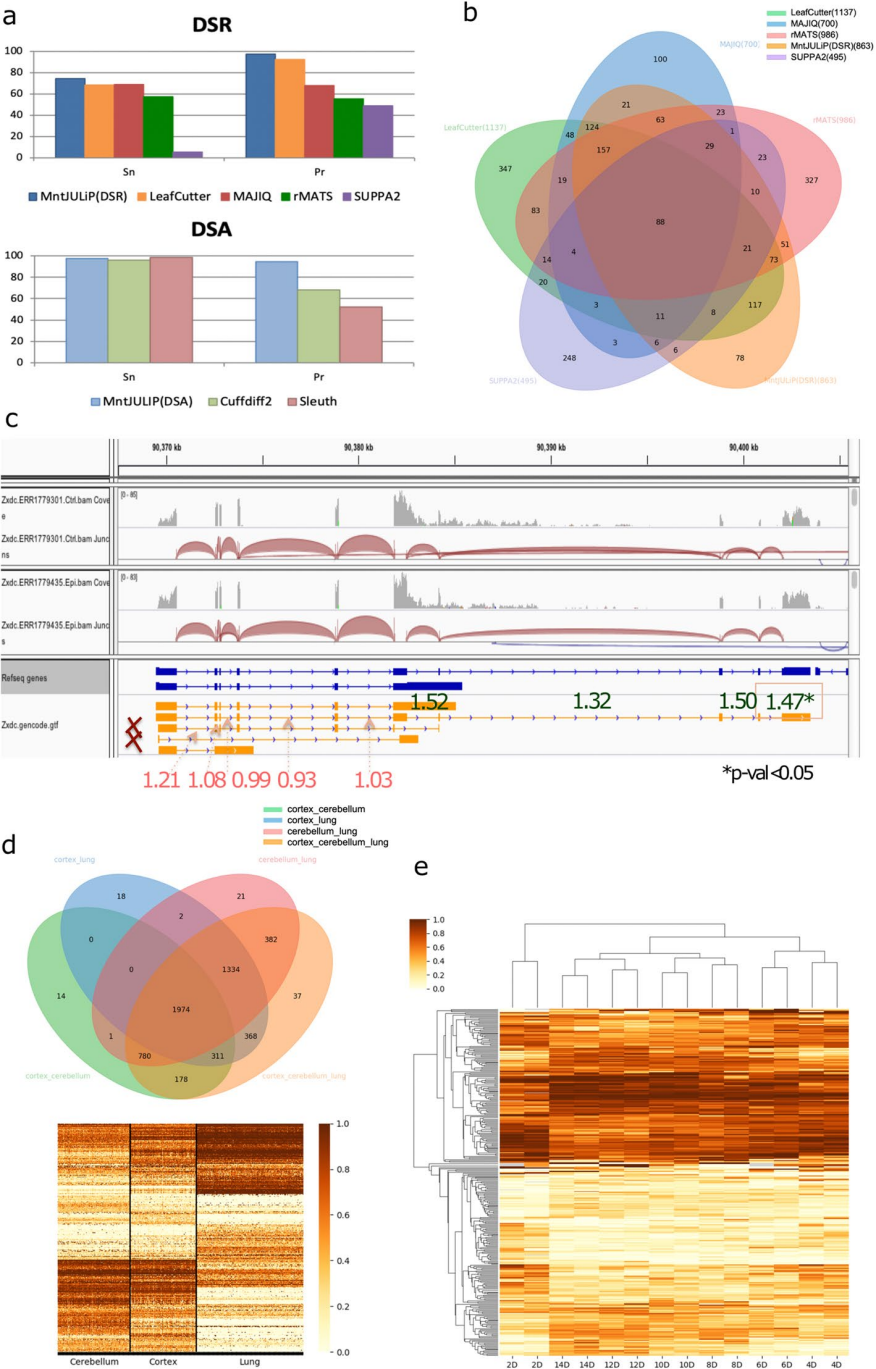
Performance evaluation of MntJULiP on simulated and real data. **A** Comparative evaluation of several methods on 25 control and 25 perturbed simulated RNA-seq data sets. **B** Venn diagram of methods’ gene-level DSR predictions on 24 healthy and 20 epileptic mice. **C** Differential splicing at the *Zxdc* gene locus discovered in the mouse hippocampus data by MntJULiP (DSA); no two introns share an endpoint; therefore, the gene could not have been discovered by other tools. Introns are annotated with the fold change values in the comparison of healthy and epileptic mice. **D** Venn diagram of DSR genes, and heatmap of DSR introns discovered with MntJULiP in a multi-way comparison of cerebellum, cortex, and lung GTEx RNA-seq samples. Rows were clustered using the Ward distance. **E** Distribution of program-predicted features by number of comparisons for three methods: (i) union of MntJULiP predicted features from all (21 total) pairwise comparisons, (ii) MntJULiP multi-way predicted features, and (iii) union of LeafCutter predicted features from all (21) pairwise comparisons. **F** Heatmap of DSR introns discovered from the multi-way comparison of 7-stage taste organoid RNA-seq data. Heatmaps show PSI values of differentially spliced introns. Clustering was performed with the Bray-Curtis distance and simple averaging

In a first, controlled experiment, we used simulated data, namely 25 control and 25 perturbed samples, to evaluate MntJULiP (DSR), MAJIQ, LeafCutter, rMATS, and SUPPA2 in detecting differences in splicing ratios, and MntJULiP (DSA), Cuffdiff2, and Sleuth in detecting differences in splicing abundance (see “Methods” and Fig. 1A). Of these, Cuffdiff2, Sleuth, and SUPPA2 rely entirely, and MAJIQ and rMATS partly, on a reference set of gene annotations to determine and quantify alternative splicing events. Further, SUPPA2 and Sleuth employ a pseudo-alignment and transcript quantification step, while the rest of the tools use genome-based read alignments, herein generated with the STAR aligner [16]. Different methods employ different target features and criteria for alternative splicing detection, some of which represent distinct splicing patterns at different locations within the gene, which makes them impossible to relate in the absence of a reference set of gene and transcript annotations. To address this limitation, we use the gene as the unit of differential splicing information. Hence, for each program tested, we consider the list of genes for which the program reports differentially spliced features and compare it to the set of simulated genes. On the DSR experiment, MntJULiP (DSR) achieved sensitivity 74.5%, which was between 8.0 and >60.0% higher than its competitors, at very high and comparable precision, 97.4%, followed by LeafCutter, at 68.4% sensitivity and 92.3% precision. On the DSA experiment, MntJULiP (DSA) had very high 97.9% sensitivity and 95.3% precision, to Cuffdiff2’s values of 95.9 and 70.3%, respectively. Sleuth marginally achieved the highest sensitivity, 98.4%, however at the expense of lower precision, 52.3%. The results ranked similarly when using an alternate aligner, Hisat2 [17] (Additional file 1: Fig. S1A). We further examined in more detail the programs’ results by gene class. While true positives for all programs were fairly uniformly distributed across the constituent gene categories, false positives for MAJIQ, rMATS, Sleuth, and Cuffdiff2 were dominated by genes outside of the simulated gene set, underscoring the difficulty for these programs to effectively distinguish and filter paralogs and other alignment and assembly artifacts (Additional file 1: Fig. S1B and Additional file 2).

Wall clock times for programs run with 10 threads, where applicable, varied between 4 and 20 min for MntJULiP, LeafCutter, SUPPA2, and Sleuth, and were significantly higher (8–9 h) for rMATS and Cuffdiff2. MntJULiP achieved the lowest estimated total “sequential” time, 46 min, with all other programs’ times varying between 5 and 84 h. Memory requirements per thread per run varied between 400 MB and 6 GB. All programs were assessed on a server with a 4 × AMD Opteron six-core CPU with 2.8 GHz and 512 GB RAM running RHEL7 (Additional file 1: Fig. S1C).

Lastly, we assessed the methods’ accuracy in quantifying the amount of change in splicing of individual introns (Additional file 1: Fig. S2). For the DSR experiment, MntJULiP predictions most closely aligned with the reference annotation (*R*^2^=0.935, Pearson correlation coefficient) between predicted and reference dPSI values, compared to 0.879 for LeafCutter and 0.847 for MAJIQ. For the DSA experiment, MntJULiP had the higher correlation (0.991 versus Cuffdiff2’s 0.848) between predicted and reference log fold change values of the two methods. Therefore, MntJULiP predicted values are strongly indicative of the amount of change and can be used reliably to inform event selection, for instance to select candidate events for experimental validation.

### Performance evaluation on real data

We next applied the methods to RNA-seq samples from hippocampus tissue of 24 healthy mice and 20 mice with pilocarpine-induced epilepsy, illustrating a typical RNA-seq experiment. Programs MntJULiP (DSR), LeafCutter, MAJIQ, rMATS, and SUPPA2 predicted between 495 and 1137 DSR genes (Fig. 1B). While it is not possible to precisely measure the prediction accuracy in the absence of a ground truth reference, we deem genes predicted by multiple tools as being more reliable. A majority of DSR genes (1024 out of 2127) were predicted by two or more tools. Importantly, MntJULiP had the smallest number and proportion of uniquely predicted genes, 78 (9.0% of its predictions), compared to 327 genes (33.2%) for rMATS, 347 genes (30.5%) for LeafCutter, 100 genes (14.3%) for MAJIQ, and 248 (50.1%) for SUPPA2, and therefore reported the smallest number of putative false positives.

DSR tests capture only a fraction of the alternative splicing variation in an experiment. To showcase the potential of MntJULiP to expand upon the classes of alternative splicing events detected, we assessed the outcomes of MntJULiP’s DSA test compared to the other methods. First, to determine whether the two tests, DSR and DSA, reveal different pathways that are impacted at the level of splicing in the induction of epilepsy, we undertook a comparative gene set enrichment analysis of the results generated by MntJULiP with the two models (Additional file 1: Fig. S3). Since the two formulations use MntJULiP’s common framework, including data models and filters, this is the first time an unbiased comparison has been performed. The comparison showed each of the DSR and DSA tests identifying unique GO categories, as well as overall differences in significance levels, pointing to differences in the regulatory pathways detected by the two methods. GO biological process categories enriched among the DSA genes involved regulation of cell migration and neuron projection, cell adhesion, and regulation of GTPase activity. In contrast, genes involved in nervous system development, including axonogenesis, synapse assembly, and neuron projection, were primarily enriched among DSR genes, with several other categories including transcription and mRNA splicing showing enrichment.

Next, we assessed the ability of MntJULiP to identify new structural classes of alternative splicing events. Of the 4187 genes predicted by MntJULiP’s DSA test, 485 were also reported by the DSR test, and an additional 443 by other tools, representing genes with traditional splicing patterns (Additional file 1: Fig. S4). An additional 2439 genes were determined to be differentially expressed by the DESeq2 [18] method, a category that is captured by the DSA test. The remaining 797 genes represent a combination of genes with traditional event patterns that could not have been discovered by other tools, and putative complex or non-conventional splicing events.

Figure 1C and Additional file 1: Fig. S5 illustrate some of these examples. The pyruvate kinase M 1/2 (*Pkm*) gene has two isoforms resulting from the use of mutually exclusive exons (Additional file 1: Fig. S5A). *Pkm1* is expressed in the adult stage where it promotes oxidative phosphorylation, whereas *Pkm2* is prevalent during embryogenesis and promotes aerobic glycolysis. Splicing dysregulation at this gene has been identified as an oncogenic driver and passenger factor in brain tumors [19]. While the difference in the isofoms’ splicing ratio is low (0.05) and may have contributed to being missed by other tools, introns flanking both exons yielded positive MntJULiP DSA tests. Most importantly, MntJULiP can detect classes of events that cannot be detected by other methods. In one example at the CWC22 Spliceosome Associated Protein Homolog (*Cwc22*) gene, the two overlapping and mutually exclusive introns at the 3′ end of the gene do not share an endpoint and therefore could not have been interrogated by other methods (Additional file 1: Fig. S5B). Similarly, none of the traditional methods can capture variation that results when one isoform’s intron chain is entirely subsumed by another, where the “extension” introns do not share endpoints with others. The ZXD Family Zinc Finger C (*Zxdc*) gene illustrates this example with its 3′ most terminal introns. The GENCODE annotation for this gene lists five isoforms, of which two can be eliminated based on the fact that their unique introns do not appear in any of the 44 samples. Of the remaining isoforms, two have their intron chains entirely subsumed by the longest isoform. In Fig. 1C, the distribution and average fold change abundance differs significantly between the shared (average 1.03) and isoform-specific (average 1.45) intron sets, which can only be explained by a difference in the proportion of splice isoforms in the gene’s output. Lastly, further case analyses revealed other intriguing scenarios, such as at the *Zfp91-Cntf* gene locus (Additional file 1: Fig. S5C). The two genes have in common the only intron in the Ciliary Neurotrophic Factor (*Cntf*) gene (chr19:12.764.380-12,765,281), which shows a significant sixfold increase in abundance in the epileptic mice, whereas all other introns for *Zfp91* show a slight decrease within statistical error. While the event can be at first sight attributed to the differential splicing of *Zfp91*, careful observation of the expressed introns reveals that the sole *Zfp91* isoform containing the intron is present at residual levels or not at all in both conditions. Therefore, the increase in abundance appears to be due to the change in the expression of *Cntf*, which owing to the special sharing of gene structure was missed by DESeq2. *Cntf* is a survival factor for multiple neuronal cell types, and an increase in its levels was shown to be involved in attenuating epilepsy-related brain damage [20, 21].

True accuracy cannot be assessed in analyses on real data. However, to evaluate robustness and reproducibility in the tools’ predictions as an alternative measure of performance [11], we divided and analyzed the data into two sets of 10 healthy and 12 epileptic mouse samples. The graphs in Additional file 1: Fig. S6 show the scatterplots of the estimated difference in percent splicing inclusion (dPSI) between the two replicated experiments. MntJULiP has the highest correlation between the runs (0.579), followed closely by MAJIQ (0.577) and LeafCutter (0.460), and therefore its results are the most robust with the sample set.

### Performance on large data sets

To demonstrate the scalability of MntJULiP and its capability to perform simultaneous multi-way comparisons, we applied it to four tissue datasets (frontal cortex, cortex, cerebellum, and lung; 554 samples total) extracted from the GTEx RNA-seq collection. We performed pairwise comparisons as well as three-way comparisons among tissues. In a first experiment comparing the three brain tissues, the multi-way comparison largely recapitulated the individual pairwise comparisons, detecting 99.0% (1070) of the 1081 genes and 11 additional genes (Additional file 1: Fig. S7A). The test also revealed highly similar splicing profiles between cortex and frontal cortex, with only one gene differentiating the samples. The robustness of the method was confirmed in a second test, comparing the cortex, cerebellum, and lung samples (Fig. 1D and Additional file 1: Fig. S7C). All but 14, 18, and 21 of the genes reported from the three pairwise comparisons were selected by the multi-way test, and 37 genes were unique to the three-way comparison, for a 99.3% (5324 out of 5364 predicted genes) recovery rate. Figure 1D and Additional file 1: Fig. S8 show the heatmaps of PSI values for each tissue and comparison, reiterating these observations. Similar results can be observed for the DSA test, where the multi-way comparison discovered 97.1% (15,090 out of 15,491) of all genes detected by pairwise comparisons, and only 36 (0.02%) unique genes among the 15,126 predicted (Additional file 1: Fig. S7D). Importantly, the comparisons highlighted thousands of differential splicing events that distinguish among the tissues [22]. Experiments took between 18 and 44 min per comparison on a 24 CPU Intel processor, thereby demonstrating the ability of MntJULiP to handle large-scale applications.

### Application to complex and time-series experiments

All differential splicing methods to date are designed for comparing two conditions, typically “cases” versus “controls.” This simple framework is inadequate and impractical for scenarios that involve time-series or complex multi-condition experiments, which seek to determine features that vary across the full range of conditions. As an illustration, we applied both LeafCutter and MntJULiP to RNA sequencing data from mouse taste organoids at seven growth stages [23] (Accession: DRA005238; two samples each at days 2D, 4D, 6D, 8D, 10D, 12D, and 14D, for a total of 14 samples). LeafCutter predicted DSR events in 889 genes and MntJULiP in 3285 genes when combining the results from all-against-all pairwise analyses. By comparison, MntJULiP’s multi-way test predicted 212 differentially spliced genes across all conditions. While true accuracy cannot be measured, we deem features (genes) reported by multiple comparisons to have higher confidence than those predicted in a single comparison, on the basis that features that are differentiated between two stages will likely show variation in other comparisons involving one of the original conditions. As Fig. 1E indicates, the distribution of genes according to the number of comparisons in which they are reported is very similar for the LeafCutter and MntJULiP pairwise protocols, with 31–36% of the genes found in only one comparison, pointing to potentially large numbers of false positives. In contrast, the distribution for MntJULiP multi-way predicted genes follows a Bell curve distribution with the mode at 8 comparisons, which provides a more realistic reflection of the experiment. Therefore, the multi-way comparison more accurately identified differences in splicing across the experimental range.

To further examine the alternative splicing variation during organoid differentiation, we generated heatmaps of the introns discovered with the MntJULiP all pairwise and the MntJULiP multi-way comparison methods (Fig. 1F and Additional file 1: Fig. S9). Introns’ PSI values show small variation in splicing between consecutive stages, but clear distinguishing characteristics when comparing across all experimental timepoints. In particular, features detected by the multi-way comparison better distinguish between the organoid growth stages, with a significant inflection point between early (days 2D–6D) and late development and differentiation into taste cells (days 8D–14D), and facilitate more accurate clustering of samples. Interestingly, the visualizations point to distinguishing features separating stage 2D from the other non-differentiated stages, and the separation becomes even more apparent in the DSA visualizations (Additional file 1: Fig. S9B). Importantly, these graphical representations highlight the ability of MntJULiP to detect even mild differences between conditions. We also note the ability of MnJULiP to work with very small numbers of samples per condition, as low as two samples per organoid stage.

### Analysis of brain GTEx data

**Fig. 2:**
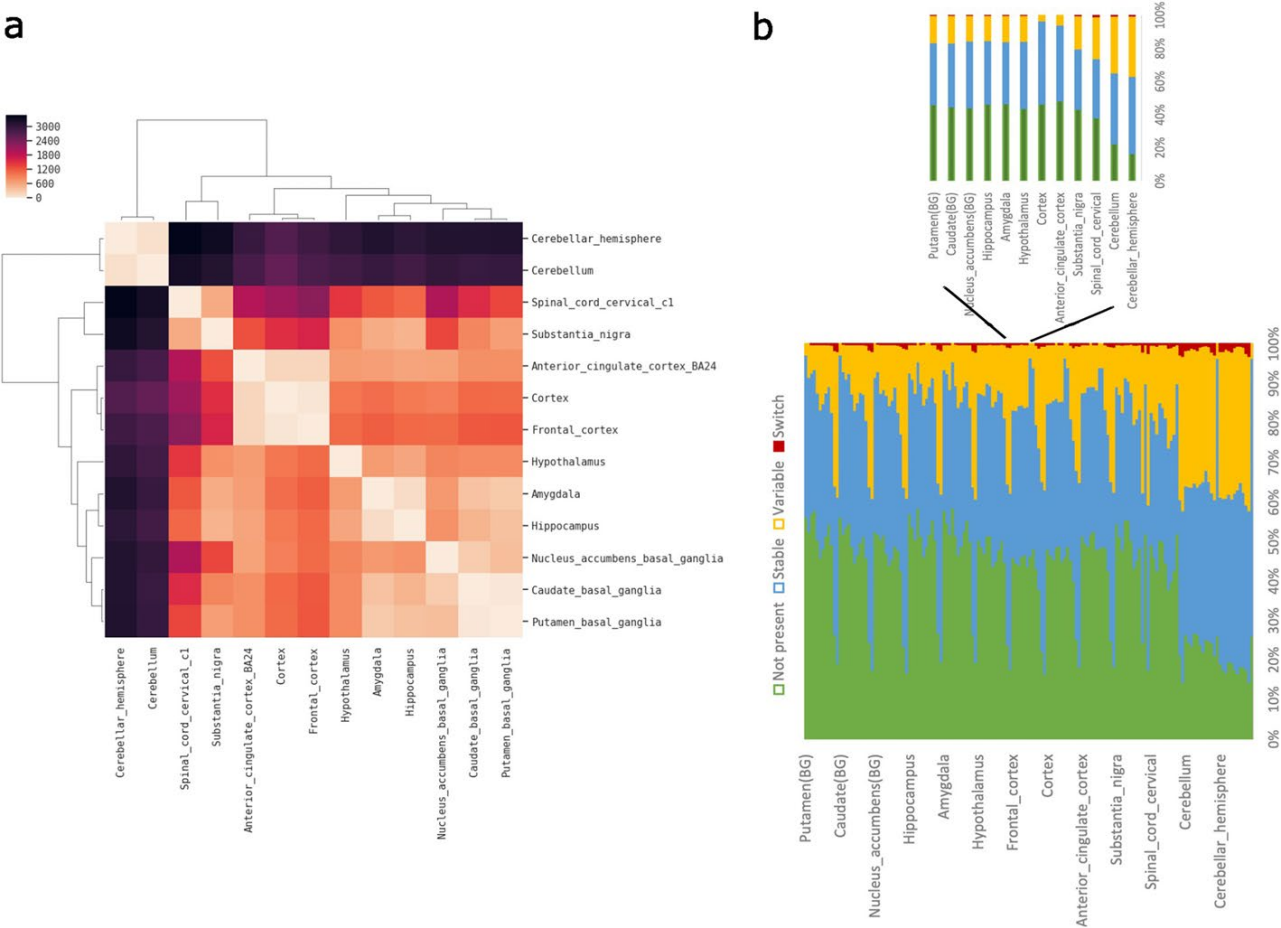
Landscape of alternative splicing variation across human brain regions from 1398 GTEx samples. **A** Dissimilarity matrix showing region-to-region splicing differences determined with MntJULiP (DSR) (*p*-value<≤0.05, |dpsi|>0.2). Clustering was performed with the Bray-Curtis distance and simple averaging. **B** Splicing patterns for the 49,186 events were compared between any two brain regions, and events were classified by the difference in the splicing ratios. The 156 × 156 matrix shows the dynamics of splicing events between one tissue and each of the others. The numbers of stable (blue), variable (gold), switch (red), and not present (green) events between any two brain regions are shown along one line

To showcase the ability of MntJULiP to perform very large-scale differential splicing analyses, we used it to analyze a collection of 1398 RNA-seq samples from 13 brain regions represented in the GTEx repository. When applied to all pairwise tissue comparisons, MntJULiP (DSR) reported differential splicing events at 426–9746 introns per comparison (*p*-val<0.05, dpsi≥0.05), occurring at 331–4745 genes, for a total of 29,347 events at 7588 genes. The simultaneous multi-way comparison of all tissues identified differential splicing events at 29,559 introns (*p*-val<0.05, dpsi≥0.05) in 6764 genes. The DSR distance matrix between tissues illustrated in Fig. 2A shows strong similarity among the three cortex regions, as well as among the basal ganglia regions, and between amygdala and hippocampus. Similarly, cerebellum and cerebellar hemisphere form a distinct group. These relationships were recapitulated when analyzing the differences in splicing abundance with MntJULiP’s DSA algorithm (data not shown).

We next examined the ability of MntJULiP to detect novel introns and novel alternative splicing events, not present in the reference annotation. Out of the 179,826 introns validated by MntJULiP across all pairwise comparisons, 14,961 were novel, not found in the GENCODE v.36 gene annotations. Of these, 5407 were reported as differentially spliced in the pairwise comparisons, and 11,242 in the multi-way comparison. We further went on to investigate the region specificity of the novel introns within the brain tissue collection, and the tissue specificity within the entirety of the GTEx data set (31 tissues) (see “Methods”). Because multiple regions may have been sampled spatially from the same tissue type and therefore have similar underlying cell type composition and transcriptional profiles, we grouped highly similar tissues and regions, in particular the cortex, basal ganglia, and cerebellar tissues, respectively. We identified 412 introns to be specific to one particular brain region, most of them to cerebellar areas (373 introns), 23 introns specific to basal ganglia, and smaller numbers (<10) to other regions. Most of these novel tissue-specific introns, 296 out of 412 (71.8%), were reported as differentially spliced by the multi-way comparison, whereas 124 (30.1%) were reported to be differentially spliced in at least one pairwise comparison. When all of the GTEx tissues were considered, 762 of the novel introns were found to be brain specific. Thus, MntJULiP has the ability to detect novel introns, in particular those distinguishing among tissues.

Lastly, we used the compendium of 49,186 alternative splicing events to draw a map of splicing variation across the human brain regions (Fig. 2B). For each pairwise comparison, we classified the change in an alternative splicing event as null or low (dpsi <0.05; “stable” events), medium (0.05≤dpsi<0.5; “variable”), and large (dpsi≥0.5; “switch”), unless the event was not present in either of the regions. Events with large effects include those that cause a major isoform switch and therefore are expected to play an important part in tissue differentiation. In Fig. 2B, a small proportion (up to 3%) of the events were “switches,” and between 2.7 and 40.2% were variable, with most events either not being present or showing little variation between pairs of regions. As noted, cerebellum and cerebellar hemisphere had a larger proportion of significant differential splicing events distinguishing them from other regions, as well as a reduced proportion of events that were not expressed, suggesting that variation in splicing patterns may contribute to a large extent to their physiological differences.

To conclude, MntJULiP is capable of analyzing very large data sets to enable the comprehensive characterization of alternative splicing variation within a tissue, organism, or species.

## Discussion and conclusions

We developed MntJULiP, a novel method that detects and quantifies alternative splicing differences at the level of introns, thus avoiding the pitfalls of short read assembly. A variety of methods for differential splicing analysis are currently available, which differ in their selection of target features, objective functions, and technical approaches, leading to poor consistency among the results they produce [15]. MntJULiP aims to provide a comprehensive view of alternative splicing variation, by representing it at the most granular level (intron) and by implementing two objective functions, aimed at determining differences in the absolute and relative (ratios) intron splicing levels. In comparisons with other programs on simulated and real data, we demonstrated that MntJULiP identifies more alternative splicing variation and more classes of variation than other tools, across a spectrum of experimental conditions, dataset sizes, and degrees of variation. Additionally, MntJULiP achieved improved accuracy, in particular precision, owing to its careful handling of low support introns, by filtering low abundance introns in individual samples and across all samples in a condition.

MntJULIp introduces several technical innovations, including its zero-inflated negative binomial and multinomial Dirichlet models to account for low count genes and splice junctions, and the mixture distributions that allow for modeling multiple conditions, thus facilitating multi-way differential analyses.

A unique capability of MntJULiP is its ability to perform multi-way comparisons, which is desirable when characterizing complex time-series or multi-condition experiments, to identify a global set of features that distinguish among subgroups or stages. Importantly, our experiments suggest that analyzing all conditions simultaneously to determine differences in the global splicing patterns increases the accuracy, in particular specificity, of results.

Lastly, MntJULiP is highly efficient and scalable and can be used with thousands of samples, providing an effective platform for comprehensive differential splicing analyses of RNA sequencing data from a wide range of experiments and data collections.

## Methods

### Algorithm overview

MntJULiP consists of two components, a “builder” and a “quantifier” (Additional file 1: Fig. S10). The *builder* extracts the splice junctions (introns) and calculates their supporting read counts from the RNA-seq read alignments, filtering introns with fewer than 3 reads in each sample, as potential sequencing and mapping artifacts. (A second filter that removes introns with weak support within the gene’s context is embedded in the statistical model below.) Individual introns are the input to the DSA analysis. For the DSR analysis, introns that share an endpoint are grouped into “bunches.” If a reference gene annotation is provided, both individual introns and bunches are associated with an annotated gene if they share at least one intron coordinate. The *quantifier* subsequently evaluates candidate introns, building a learning model for each intron and bunch and performing two statistical tests: (i) a test for change in intron abundance (*DSA*), and (ii) a test for change in the splicing level of the intron relative to its “bunch” (*DSR*). For the DSA analysis, MntJULiP uses a mixture zero-inflated negative binomial model to estimate individual introns’ abundance levels from the raw read counts. For DSR, it estimates the relative splicing ratios with a mixture Dirichlet multinomial distribution. For both models, likelihood ratio tests are used to determine the differential splicing events and generate *p*-values, which are then adjusted for multiple testing using the Benjamini-Hochberg correction. The framework is described in detail below.

### A Bayesian read count model

We use a Bayesian statistical framework to estimate the splicing levels of introns for differential analyses. The framework also provides a second filter for weakly supported introns within the context of the gene, by setting a cutoff value for the estimated read count mean. To start, we assume that the read count *y* of intron *v* in a given sample follows a negative binomial distribution *NB*(*μ*, *θ*). We consider a loose prior with an empirical $\hat{\mu}$ (the sample mean) modeled by a normal distribution: $\mu \sim N\left(\hat{\mu},\sqrt{\frac{\hat{\mu}}{10}}\right)$ to model the variability between conditions and among the individual samples. Additionally, we apply a restriction on the dispersion parameter with an inverse Half-Cauchy distribution: *φ*^−1^~*HC*(0, 5). Lastly, to model low expression introns (0 reads in most samples), we use a zero-inflated enhanced negative binomial Bayesian model [24]:

$$y\sim \left\{\begin{array}{c}0,\mathrm{with}\ \mathrm{probability}\ \pi \\ {}\ NB\left(\mu, \theta \right),\mathrm{with}\ \mathrm{probability}\ \left(1-\pi \right)\end{array}\right.$$

Let *p*(*y*) denote the probability density function for this model. For *n* samples and intron read count *y*_*j*_ in sample *j*, we define the log likelihood:

$$L\left(\theta \right)=\log p\left({y}_1,{y}_2,\dots, {y}_n\right)=\sum_{j=1}^n\log p\left({y}_j\right)$$

We maximize the log likelihood function using the Limited-memory Broyden–Fletcher–Goldfarb–Shanno (LM-BFGS) optimization method and obtain point estimates for parameters *μ*, *θ* over the samples.

### The differential splicing abundance (DSA) model

The previous section established the general Bayesian model to estimate intron abundance. Next, we describe the framework for modeling individual intron abundance and for DSA testing in a *multi-condition* experiment. Assume that samples are drawn from *m* (typically 2) conditions. Given an intron *v* and a sample generated from condition *i*, the intron’s read count *y* follows a zero-inflated negative binomial distribution with the condition-specific parameters *μ*_*i*_, *θ*_*i*_, *φ*_*i*_ , and *π*_*i*_, as defined earlier.

Let *p*_*i*_(*y*) be the probability density function for the complete model for condition *i* = 1…*m*. We define a mixture probability model for *y*:

$$\overline{p}(y)=\prod_{i=1}^m{p}_i{(y)}^{z_i}$$

where *z*_*i*_ is the indicator variable for that sample, equal to 1 iff the sample belongs to condition *i* and 0 otherwise.

To formulate the problem, given *n* samples, *m* conditions, and *y*_*j*_ the intron read count in sample *j* = 1…*n*, we define the log likelihood:

$$L\left(\theta \right)=\log \overline{p}\left({y}_1,{y}_2,\dots {y}_n\right)=\sum_{i=1}^m\sum_{j=1}^n{z}_{ij}\log {p}_i\left({y}_j\right),$$

with *z*_*ij*_ ∈ {0, 1} the indicator variable for sample *j* and condition *i*.

Having these two Bayesian models, we establish a hypothesis test for differential intron abundance given the data: the null hypothesis is that samples are generated from the same condition, and the alternative hypothesis is that the samples belong to different conditions, and apply a likelihood ratio test:

$$LR=-2\left[L\left({\theta}_0\right)-L\left({\theta}_1\right)\right]$$

where *L*(*θ*_*o*_), *L*(*θ*_1_) are the log likelihoods of the null and alternative hypothesis models, respectively, with parameters *θ*_0_ and *θ*_1_.

Lastly, since the parameter *μ*_*j*_ of the alternative hypothesis model is the expected read count (mean) of the intron in condition *j*, we can establish an additional intron filter by setting a threshold for *μ*_*j*_ (e.g., *μ*_*j*_ ≥ 1), to separate a “true” intron from “noise.”

### The differential splicing ratio (DSR) model

We next formulate the framework to test for differences in splicing ratios of introns within a “bunch,” i.e., group of introns sharing an endpoint. For simplicity, we start by assuming that all samples belong to the same condition and the read counts *y*_1_, *y*_2_, …, *y*_*k*_ in a bundle with *k* introns follow a Dirichlet multinomial distribution with priors *α*_1_, *α*_2_, …, *α*_*k*_ : *y*_1_, *y*_2_, …*y*_*k*_~*DM*(*α*_1_, *α*_2_, …, *α*_*k*_).

Let *p*(*y*_1_, *y*_2_, …, *y*_*k*_) be the probability density function of the Dirichlet multinomial distribution. For intron read counts *y*_*j*_ = (*y*_1*j*_, *y*_2*j*_, …, *y*_*kj*_) in sample *j* = 1…*n*, we define the log likelihood function:

$$L\left(\theta \right)=\log p\left({y}_1,{y}_2,\dots, {y}_n\right)=\sum_{j=1}^n\log p\left({y}_j\right)$$

Similar to the discussion in the previous subsection, to extend to the case where samples belong to multiple conditions, we define a Dirichlet multinomial distribution with prior *α*_*i*1_, *α*_*i*2_, …, *α*_*ik*_ for each condition *i* = 1…*m*:

$${y}_1^i,{y}_2^i,\dots, {y}_k^i\sim DM\left({\alpha}_{i1},{\alpha}_{i2},\dots, {\alpha}_{ik}\right)$$

Let *p*_*i*_ = (*y*_1_, *y*_2_, …, *y*_*k*_) be the probability density function for condition *i*. We define the log likelihood function:

$$L\left(\theta \right)=\log p\left({y}_1,{y}_2,\dots, {y}_n\right)={\sum}_{i=1}^m{\sum}_{j=1}^n{z}_{ij}\log {p}_i\left({y}_j\right)$$

where *y*_*j*_ = (*y*_1*j*_, *y*_2*j*_, …, *y*_*kj*_) are the read counts of introns in this bunch in sample *j*, *z*_*ij*_ ∈ {0, 1} indicates whether sample *j* belongs to condition *i* or not, and *θ* represents the parameter set of the model.

With the two Bayesian models above, we formulate a log likelihood ratio test as before: the null hypothesis assumes all samples belong to the same condition, and the alternative hypothesis assumes multiple conditions. Under the alternative hypothesis, the parameters *α*_*i*1_, *α*_*i*2_, …, *α*_*ik*_ for condition *i* can be used to define the splicing ratio, similar to Percent Splicing Inclusion (PSI) [10, 25], Ψ_*il*_ for intron *l* = 1…*k* under condition *i*, as:

$${\Psi}_{il}=\frac{\alpha_{il}}{\sum_{l^{\prime }=1}^k{\alpha}_{il\prime }}$$

### Sequences and materials

#### Simulated data

We generated 25 control and 25 perturbed RNA-seq samples with ~86 million 101 bp paired-end reads each, using the software Polyester [26] with human GENCODE v.22 as reference annotation. For the control samples, we used a model of gene and transcript abundance inferred from lung fibroblasts (GenBank Accession: SRR493366). To simulate the perturbed condition, we randomly selected 2000 annotated protein coding genes with two or more expressed isoforms and assigned them to four groups as follows [12, 27]: (i) 500 genes were left unperturbed (NONE); (ii) 500 genes had only expression changes (DE), where genes were randomly assigned one half or double the original FPKM value; (iii) 500 genes had only splicing differences (DS), obtained by swapping the expression values of the top two isoforms; and (iv) 500 genes had both expression and splicing changes (DE-DS). Thus, 1500 genes underwent changes in splicing isoform abundance, and 1000 had differences in splicing and were used as the gold reference for evaluating the tools under the DSA and DSR models, respectively.

#### Real data

Reads for 44 mouse hippocampus samples (24 cases and 20 controls) were obtained from GenBank (ProjectID: PRJEB18790). Tissue RNA-seq samples for comparative analyses (121 cortex, 105 frontal cortex, 132 cerebellum, and 196 lung samples) were obtained from the GTEx collection [28]. Lastly, RNA-seq data from differentiating mouse taste organoids [23] (14 samples, 7 stages) were obtained from the Sequence Read Archive (Accession: DRA005238).

### Performance evaluation

Reads were mapped with the program STAR v.2.4.2a [16], and separately with Hisat2 v2.2.1 [17], to the human genome GRCh38 or mouse genome GRCm38 (mm10), as applicable. Alignments were analyzed with the programs MntJULiP v1.0.0, LeafCutter v0.2.8, MAJIQ v1.1.7a, rMATS v3.2.5, SUPPA2 v2.3, Sleuth v0.30.0, and Cuffdiff2 v2.2.1 to determine changes in alternative splicing profiles. For the simulated tests, transcripts were reconstructed across each sample with StringTie v2.1.4 then merged across samples with StringTie (ST)-merge and the GENCODE transcripts as reference, to create a set of gene annotations to be used with all programs. Transcript expression levels were estimated with salmon v1.3.0 [29] for SUPPA2, and with kallisto v0.48.0 [30] for Sleuth. To evaluate the programs’ accuracy in predicting differentially spliced genes from the simulated data, the 1000 (DS, DE-DS) gene set and the 1500 (DS, DE, DE-DS) gene set were used as the gold standard for DSR and DSA prediction, respectively. Any other program predictions were deemed false positives. Standard sensitivity (Sn = TP/(TP+FN)), precision (Pr = TP/(TP+FP)), and the F1 = 2×Sn×Pr/(Sn+Pr) value were used to measure accuracy. To assess the programs’ fidelity in quantifying alternative splicing for the DSR test, reference Percent Splice Inclusion (PSI) values for all reference introns were calculated from the simulated data, as the ratio between the intron abundance and that of its bunch. Similarly, for the DSA test, reference log fold change values were calculated for each intron as the log fold change of the cumulative expression levels of all splice isoforms containing that intron.

### Functional analysis

Gene set enrichment analyses of the differentially spliced genes identified by MntJULiP DSA and DSR in the comparison of epileptic and healthy mouse hippocampus tissue was performed with the tool GeneSCF [31]. The analysis was performed on all GO databases (db=GO_all option) and the background was set to 20,000 genes (bg=20000 option). Gene symbols were used for input (-t=sym) and the organism option was set to “mouse.” The lists of enriched GO categories were further summarized using the online tool Revigo [32].

### Analysis of GTEx brain samples

Read alignments for 1398 GTEx samples from 13 brain regions (“amygdala,” “anterior cingulate cortex,” “caudate basal ganglia,” “cerebellar hemisphere,” “cerebellum,” “cortex,” “frontal cortex,” “hippocampus,” “hypothalamus,” “nucleus accumbens basal ganglia,” “putamen basal ganglia,” “spinal cord cervical c1,” and “substantia nigra”) were generated with Hisat2 v2.2.0 and analyzed with MntJULiP v1.0.0. All 78 pairwise comparisons and the 13-way comparison were performed. To determine novel introns, introns that passed the initial MntJULiP filters and were included for examination with the DSR and DSA tests were considered and were compared to the collection of GENCODE v.36 annotated introns. To determine tissue specificity, we employed the following procedure. An intron was determined to be “present” in one “tissue” if it was present (>10 reads) in 15% or more of the samples for that “tissue.” For each intron, a matrix of expected (E), defined as 0.85 × the number of samples, and observed (O) number of samples in which the intron was absent, for all introns where O≥E, was generated and analyzed with a chi-square test, to weed out any introns that are “absent” at the margin of statistical error. Lastly, introns that were “present” in exactly one “tissue” and that passed the statistical “absence” test (*p*-value≤0.001) were deemed as specific to that particular “tissue.” For the brain region specificity analysis, we merged the cortex (3), basal ganglia (3), and cerebellar (2) regions, respectively, into a single “tissue” for each, as described in [33]. For the GTEx-wide tissue specificity analysis, intron count data for the 14,961 novel introns were extracted from Snaptron [33] using a local script, and samples were organized in the 31 tissues based on their GTEx tissue assignation.

### Visualization

Heatmaps, Venn diagrams, and other graphical displays were produced using the visualization toolkit Jutils [34] and custom Python scripts.

## Supplementary Information

Additional file 1. Supplementary figures. Supplementary Figures S1-S10.

Additional file 2. Source data for figures. Supporting data for Figure 1A,E and Figure 2A,B.

Additional file 3. Listing of commands used in the analyses.

Additional file 4. Peer review history.

## Data Availability

Raw sequence data is publicly available from GenBank (ProjectID: PRJEB18790, mouse hippocampus data; and Sequence Read Archive Accession: DRA005238, organoid data), and from the GTEx repository. The MntJULiP software is available from GitHub [35] and from the Zenodo repository [36]. Alignments of simulated data, scripts, and results from all analyses can be obtained from the MntJULiP GitHub site and from Zenodo [35, 37]. Additional figures and tables, and supporting data, are available as supplementary material included with this manuscript (Additional file 1, Additional file 2 and Additional file 3).
