# Supplementary material for: Comprehensive and scalable quantification of splicing differences with MntJULiP
Source: Genome Biol. 2022 Sep 14;23:195. doi: 10.1186/s13059-022-02767-y (PMC9472403; doi:10.1186/s13059-022-02767-y)

SUPPLEMENTARY MATERIAL FOR THE MANUSCRIPT:

“COMPREHENSIVE AND SCALABLE QUANTIFICATION OF SPLICING DIFFERENCES WITH MNTJULIP”

by: Guangyu Yang, Sarven Sabunciyan, and Liliana Florea

Table of Contents:

**Supplementary Figures and Tables**

**Fig S1.** Performance evaluation of programs in the simulation experiment.

**Fig S2.** Quantification accuracy of programs at intron level in the simulation experiment.

**Fig S3.** Comparative gene set enrichment analysis of MntJULiP DSA and DSR results for mouse hippocampus data.

**Fig S4.** Venn diagram of programs’ predictions on the mouse hippocampus data set.

**Fig S5.** Examples of MntJULiP predictions not identified with other tools (mouse hippocampus data set).

**Fig S6.** Reproducibility plots for MntJULiP, LeafCutter and MAJIQ (DSR test) and MntJULiP (DSA test) on the mouse hippocampus data.

**Fig S7.** Multi-way versus all-against-all pairwise comparisons on GTEx tissue samples - gene sets.

**Fig S8.** Multi-way versus all-against-all pairwise comparisons on GTEx tissue samples - heatmaps.

**Fig S9.** Heatmaps of differentially spliced features in the taste organoid data set.

**Fig S10.** Overview of the MntJULiP algorithm.

**Fig S1.** **Performance evaluation of programs in the simulation experiment.** (A) Performance with alternate aligner (Hisat2). Note, SUPPA2 and Sleuth have built-in pseudo-alignment and transcript quantification steps, rather than using genome-based alignments, and their results remain unchanged. **(B)** Predictions by gene category. Breakdown of programs’ predictions by the four gene categories (DS, DE, DE-DS and NONE), and novel (Others), i.e. not in the simulation set. (C) Runtime and memory evaluation of programs on the simulated data set.

(A)


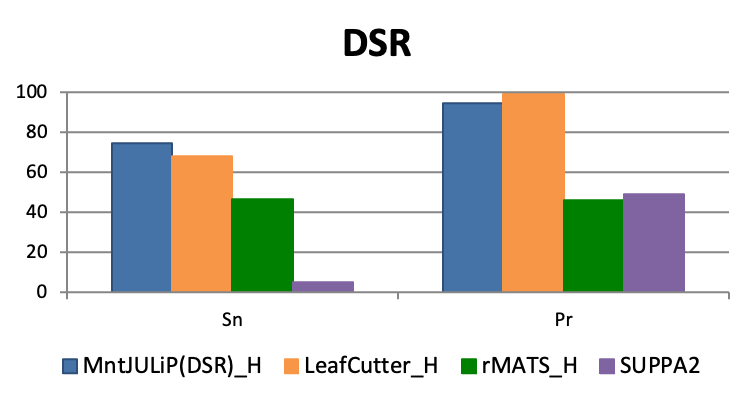

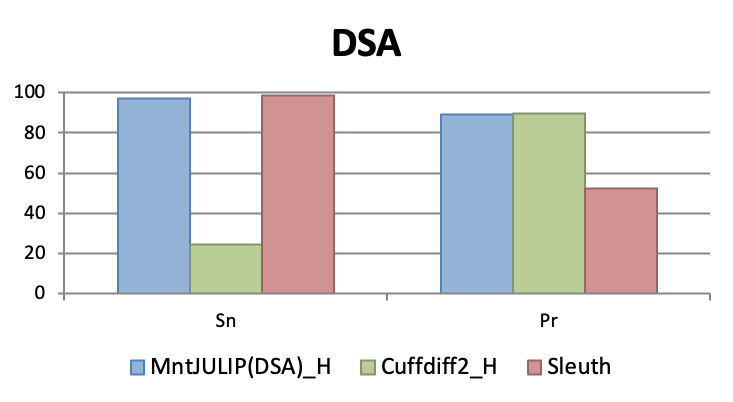


(B)


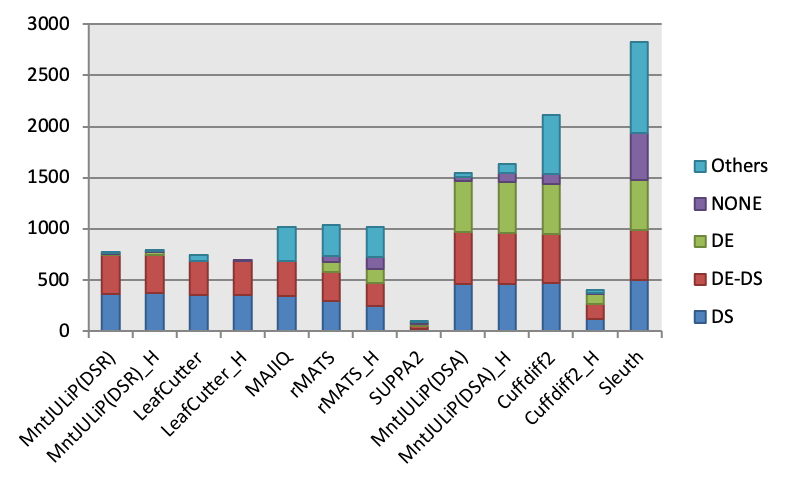


**(C)**

**Fig S2.** **Quantification accuracy of programs at intron level in the simulation experiment**. (A) *DSR test:* Scatterplots of reference and predicted dPSI = PSI_test_-PSI_ctrl_ values for 11,282 (MntJULiP), 12,023 (LeafCutter) and 23,406 (MAJIQ) reported introns. Red points mark predictions with p-val<=0.1 and |dPSI|>=0.05. (B) *DSA test:* Scatterplots of reference and predicted log2fc = log_2_ (N_test_/N_ctrl_) values for 31,868 reported introns (MntJULiP) and 10,305 (Cuffdiff2) isoforms. The p-val=0.1 line corresponding to the programs’ prediction cutoff is marked with a dotted line. (C) Table of Pearson correlation coefficients for the DSR and DSA scatterplots above, considering all introns (isoforms, for Cuffdiff2) evaluated by a program and introns (isoforms) correctly predicted by the program to be differentially spliced (TPs), respectively. MntJULiP shows the highest correlation in each category.

(A)


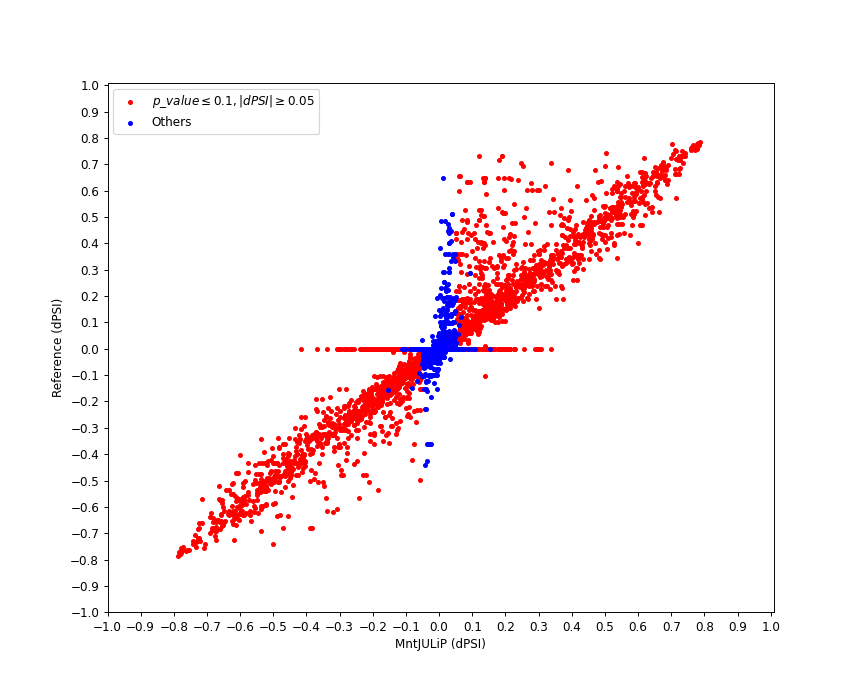

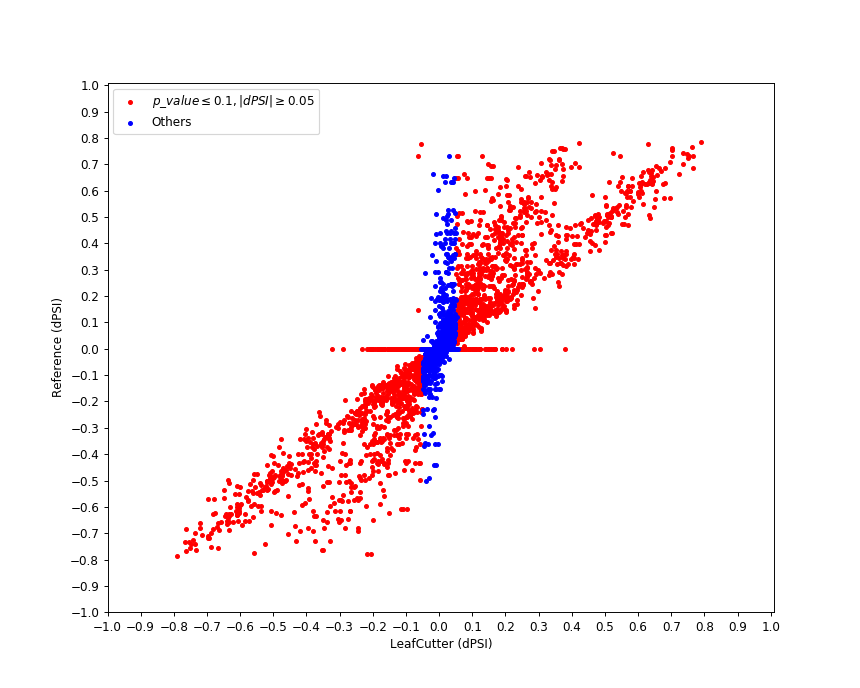


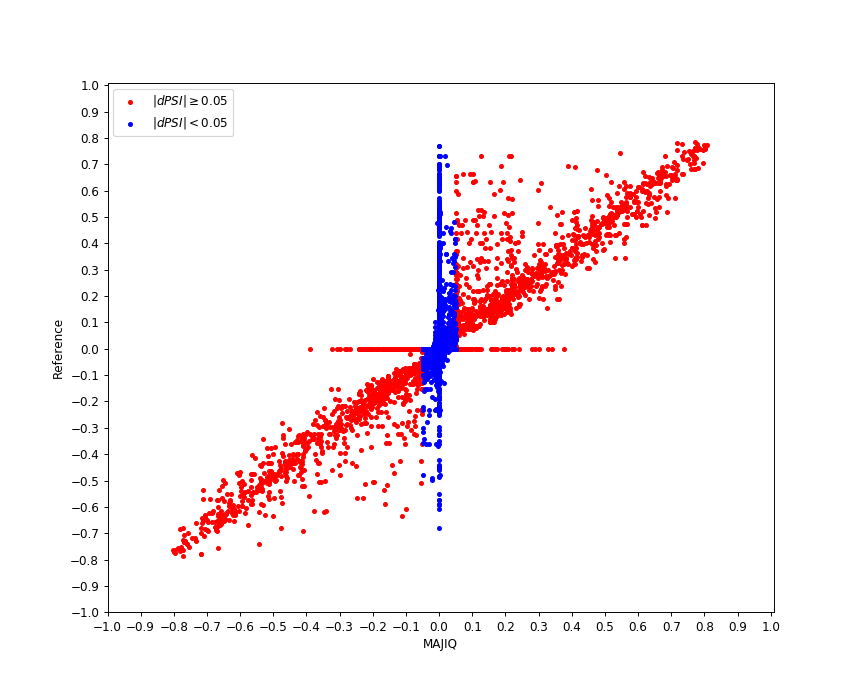


(B)


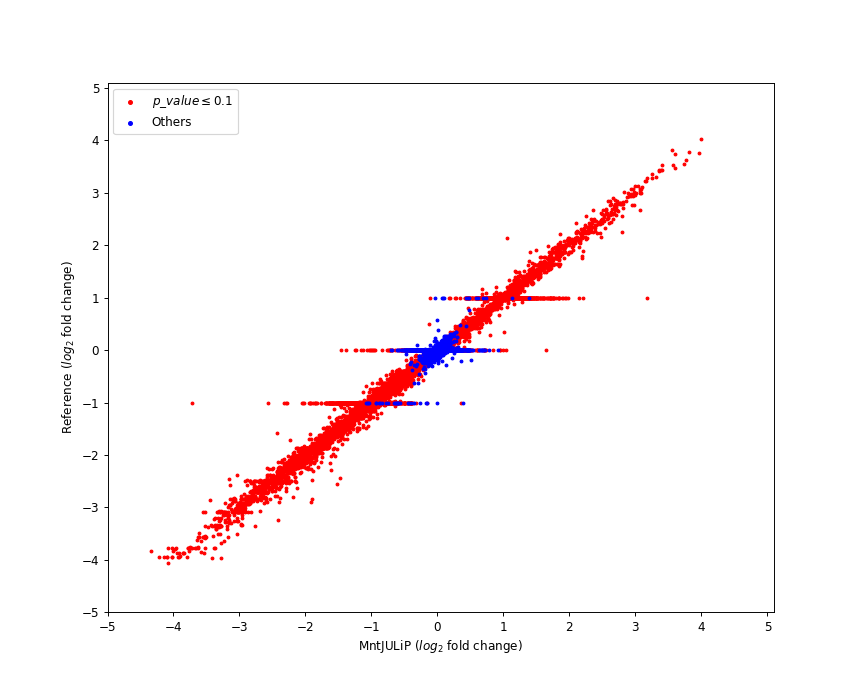

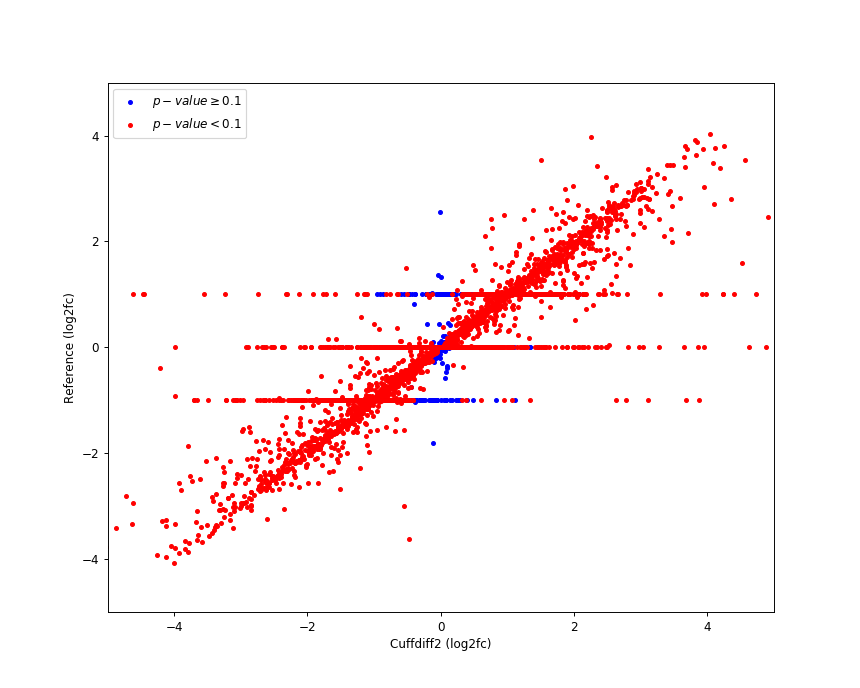


(C)

**Fig S3.** Comparative gene set enrichment analysis of MntJULiP DSA and DSR results for mouse hippocampus data. (A) Scatterplots of p-values of GO biological process (BP) categories for categories enriched among DSR (left) and DSA (right) reported genes, respectively. Red marks – categories significant in both comparisons (p-val<=0.05); blue marks – categories significant only in the featured comparison. (B) Enriched GO BP categories summarized and visualized with the tool Revigo (Supek *et al.*, *PLoS ONE* 2011). Color is used to indicate significance as reflected by summarized p-values, and the size of the markers indicates the frequency (generality) of the term(s) in the Gene Ontology database. For DSR results (left), categories associated with nervous systems development are the most significant (purple). For DSA results (right), categories clustered under positive regulation of gene expression and regulation of GTPase activity are among the most significant. (C,D) Detailed scatterplot representations and listings of Revigo summarizations for the DSR, DSA comparisons.

(A)

(B)

(C)**
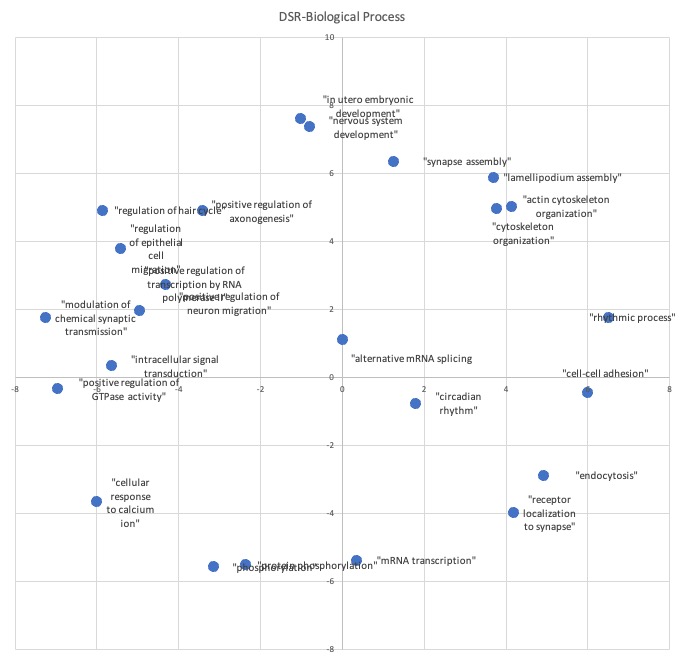
**

**(D)**

**
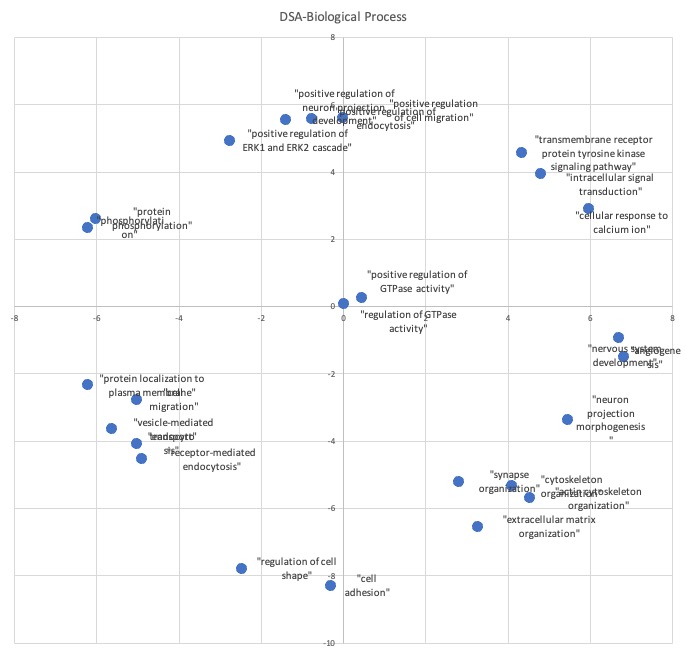
**

**Fig S4.** Venn diagram of programs’ predictions on the mouse hippocampus data set.

**
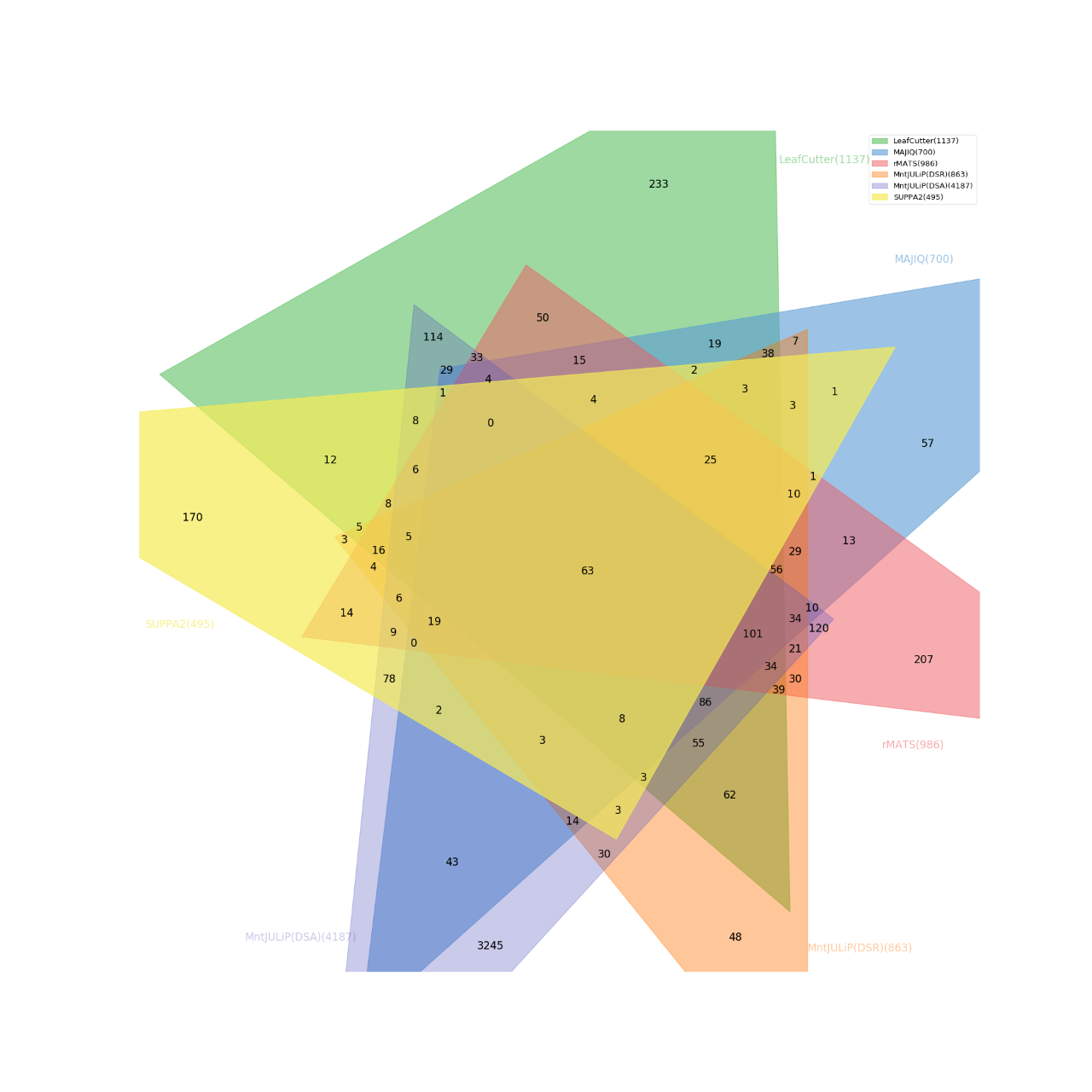
**

**Fig S5.** Examples of MntJULiP DSA predictions not identified by other tools (mouse hippocampus data set). (A) A mutually exclusive exon event at the *Pkm* gene locus is missed by DSR tools, but is identified by MntJULiP’s DSA test via two of the flanking introns with significant changes in abundance. (B) The gene *Cwc22* harbors two overlapping and therefore mutually exclusively used introns, chr2:77881490-77903814 and chr2:77896578-77903796, with the former identified as significantly differentially abundant. (C) The gene *Cntf* shares its single intron (chr19:12,764,380-12,765,281) with the *Zfp91* gene. The intron is predicted by MntJulip DSA to undergo significant changes in abundance. The only *Zfp91* isoform containing this intron can be excluded based on the fact that one or more unique introns are not represented in the alignment data, therefore pointing to the gene *Cntf* being differentially expressed (*n.b.*, the differential expression of the gene *Cntf* was missed by DESeq2 due to its structure overlap with *Zfp91*). Note that the genes in (B) and (C) do not have *any* endpoint sharing introns, which is the required pattern for DSR-based methods, and therefore are *not* identifiable by any of the other tools.

(A)

(B)

(C)

**Fig S6.** Reproducibility plots for MntJULiP, LeafCutter and MAJIQ (DSR test) and MntJULiP (DSA test) on the mouse hippocampus data (A-D). Mouse hippocampus samples were divided randomly into two sets of 10x12 samples (healthy versus epileptic) each, and the per intron dPSI values (log2fc) predicted by each program are plotted between the two comparisons. Number of introns represented: 16,607 for MntJULiP (DSR), 30,738 for LeafCutter, 93,642 for MAJIQ, and 132,289 for MntJULiP (DSA). Correlation coefficients for the 4 comparisons are 0.579 for MntJULiP (DSR), 0.460 for LeafCutter, 0.577 for MAJIQ, and 0.665 for MntJULiP (DSA).

(A) (B)


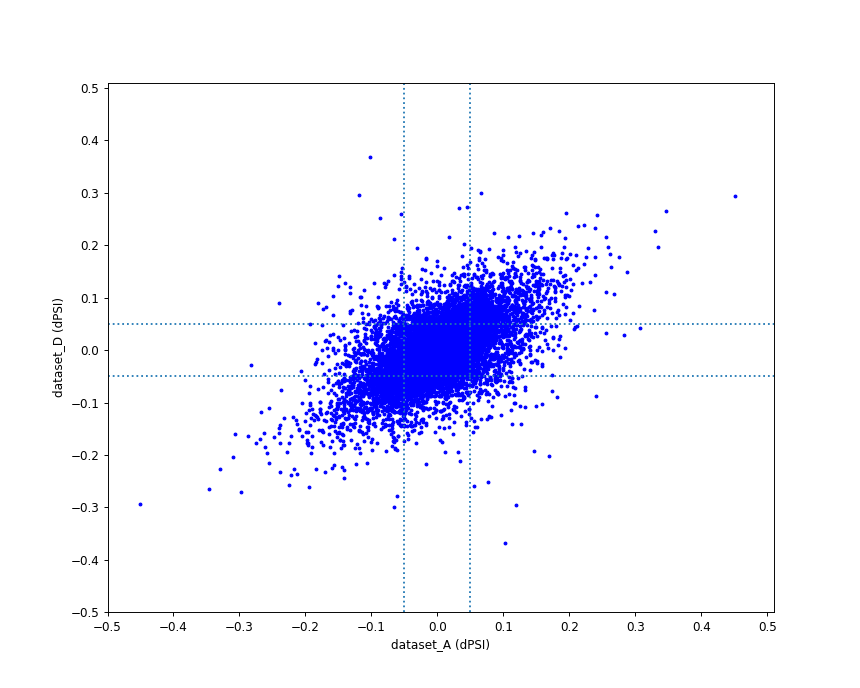

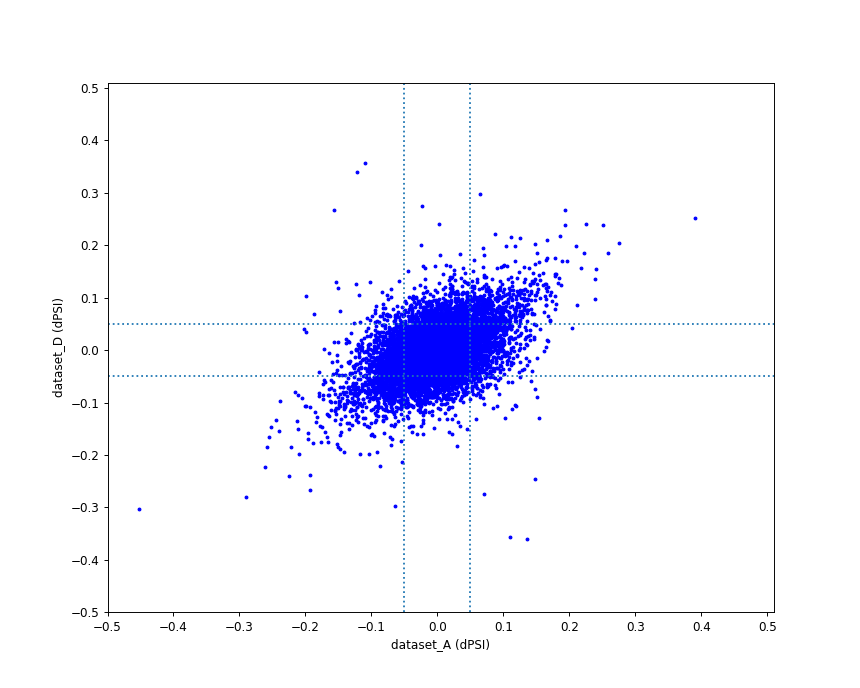


(C) (D)
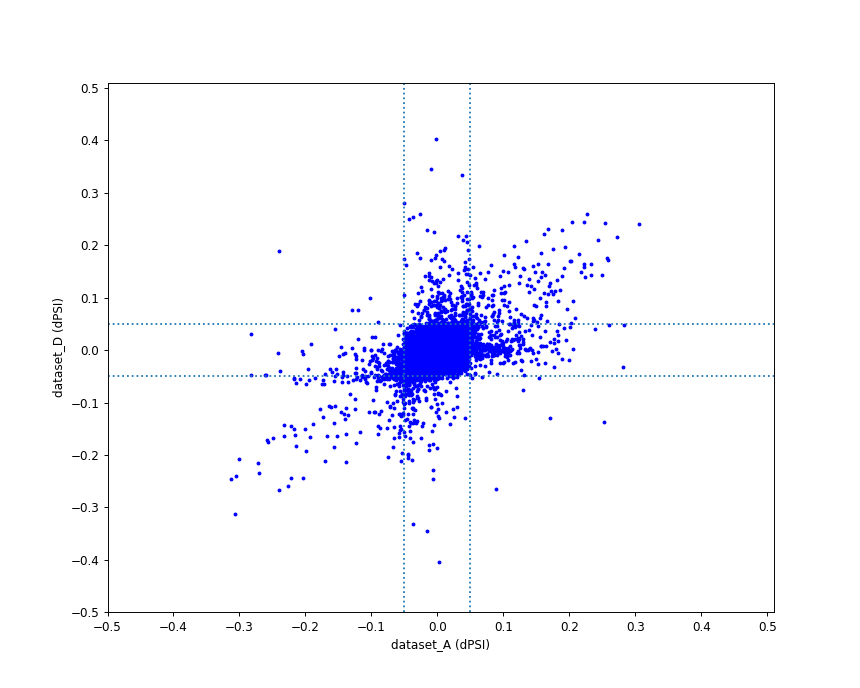

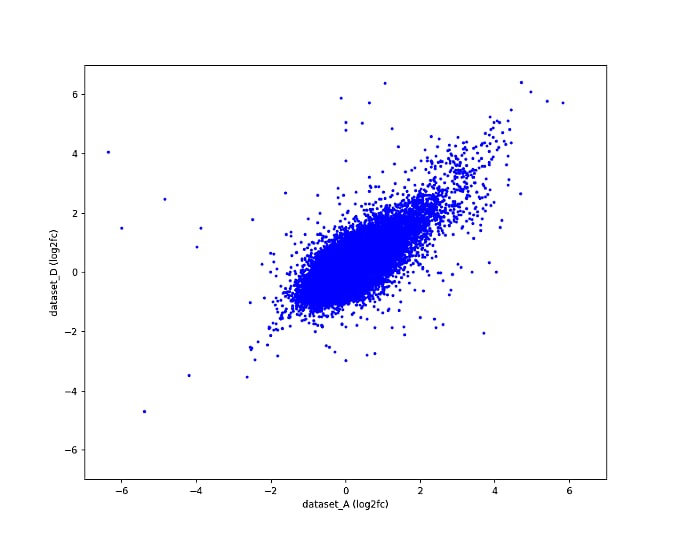


**Fig S7.** Multi-way versus all-against-all pairwise comparisons on GTEx tissue samples - gene sets. (A-B) Venn diagram of gene sets predicted by MntJULiP DSR (DSA) in comparisons of frontal cortex (105 samples), cortex (121 samples) and cerebellum (132 samples) RNA-seq collections. (C-D) Venn diagram of gene sets predicted by MntJULiP DSR (DSA) in comparisons of cortex (121 samples), cerebellum (132 samples) and lung (196 samples) RNA-seq collections. (A p-value cutoff of 0.05 was used for all comparisons.)

(A) (B) **
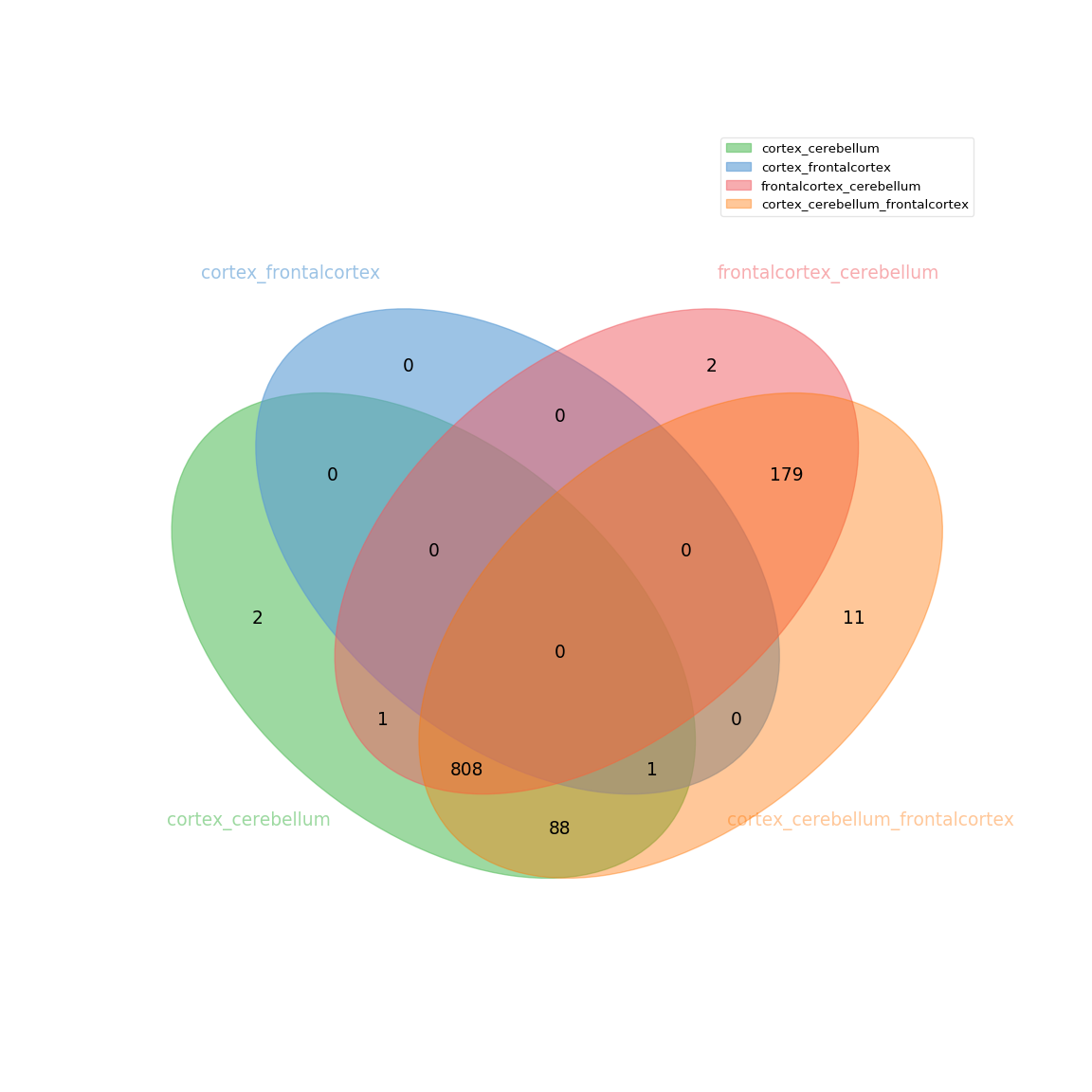
**
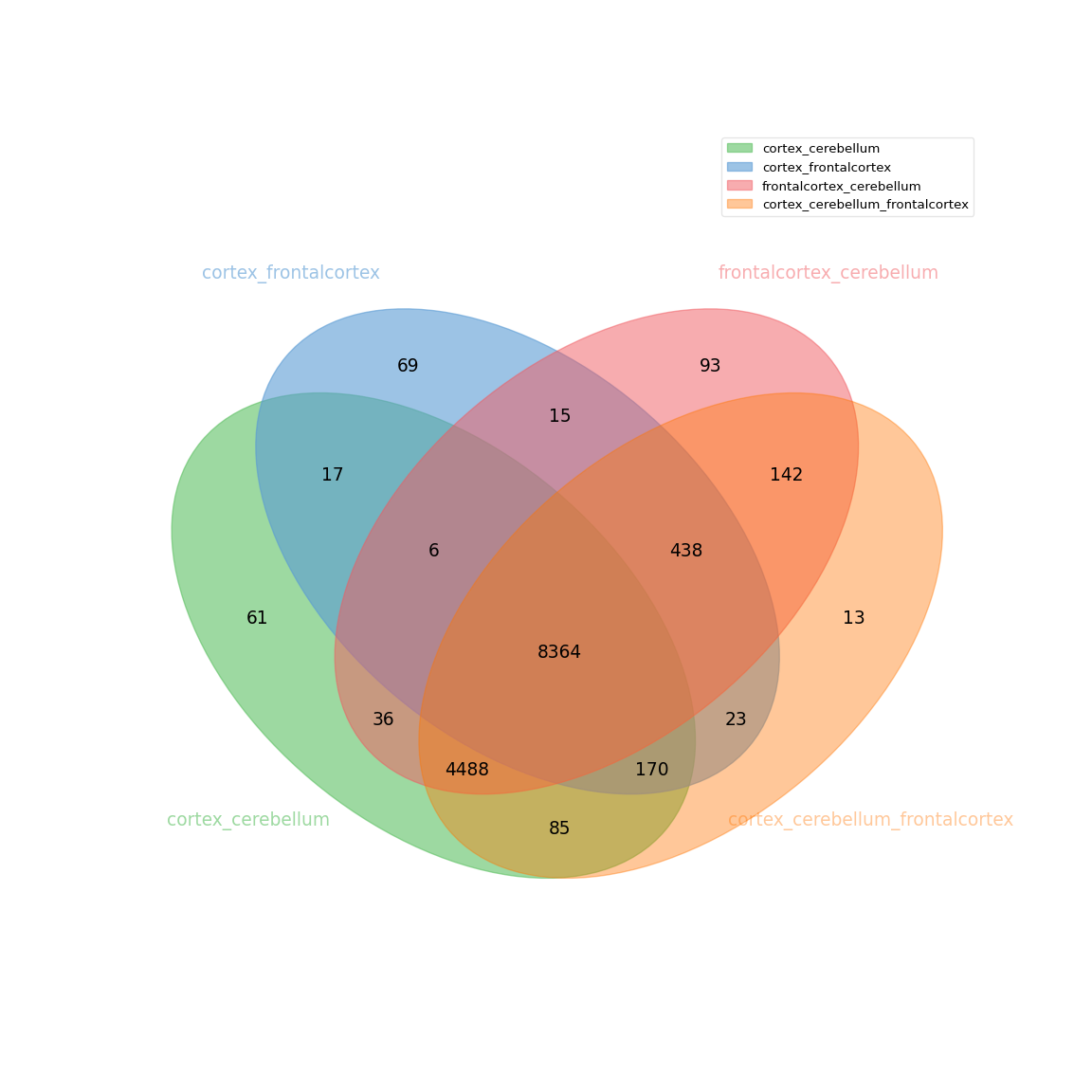


(C) (D)

**
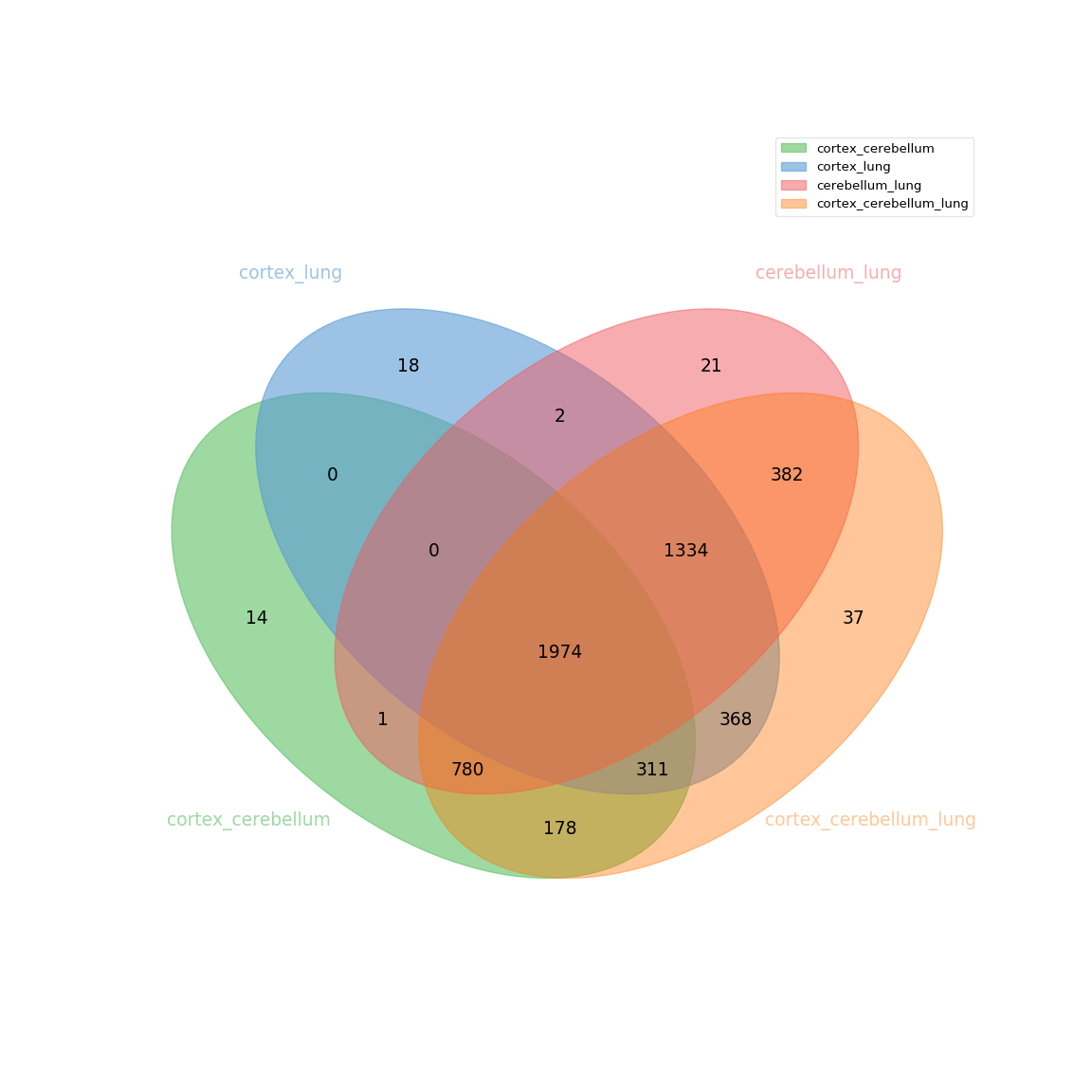

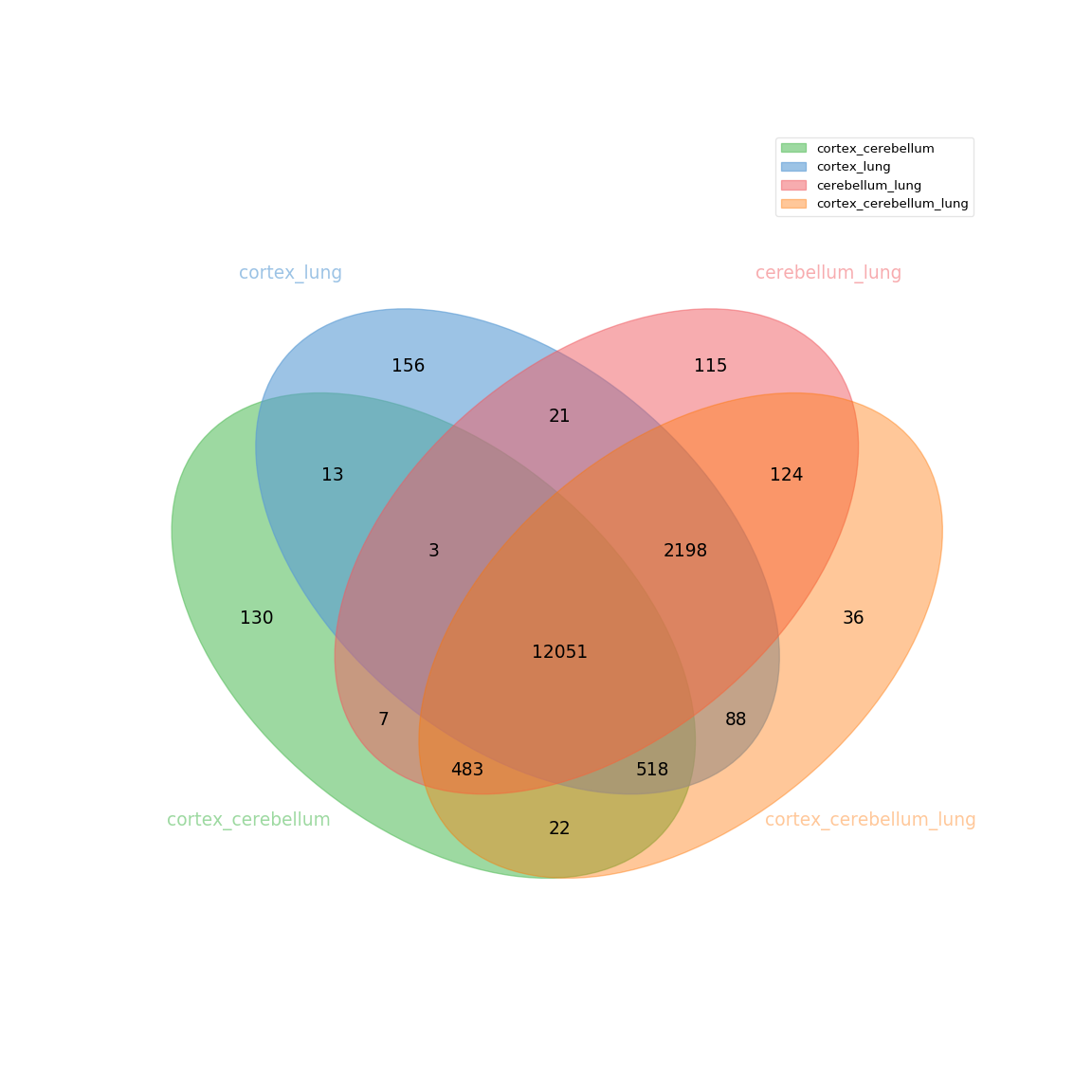
**

**Fig S8.** Multi-way versus all-against-all pairwise comparisons on GTEx tissue samples - heatmaps. (A) Heatmap of MntJULiP-identified DSR introns for the three-way and pairwise comparisons of frontal cortex, cortex and cerebellum collections. (B) Heatmap of MntJULiP-identified DSR introns in the three-way comparison of cortex, cerebellum and lung data collections. (C) Heatmap of MntJULiP-identified DSA introns for the three-way and pairwise comparisons of frontal cortex, cortex and cerebellum data collections. (D) Heatmap of MntJULiP-identified DSA introns in the three-way comparison of cortex, cerebellum and lung data collections. Note: Introns from genes with >30 reads across all samples, with dPSI>=0.2 (for DSR) and q-value<0.05, were plotted. For DSA, additionally, only the intron with the largest log 2 fold change was chosen to represent the gene.

(A)

(B)

**
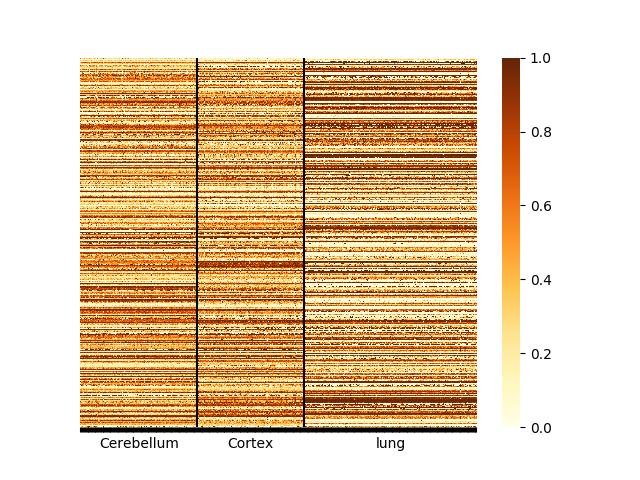
**

(C)

(D)


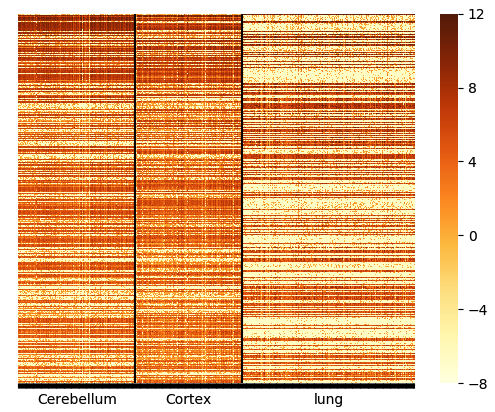


**Fig S9.** Heatmaps of differentially spliced features (introns) in the taste organoid data set. (A) MntJULiP DSR, features discovered via all pairwise versus multi-way comparison: (left) ‘all pairwise’ features, heatmap clustered by rows and columns; (center) ‘multi-way’ features, heatmap clustered by rows and columns; (right) ‘multi-way’ features, clustered by row (feature), samples ordered according to the time series. (B) Similarly, for MntJULiP DSA-predicted features. Features were filtered at p-value<0.05 and dPSI>=0.2 (for DSR). Grouping was performed using weighted hierarchical clustering with the Bray-Curtis metric.

(A)

(B)

**Fig S10.** Overview of the MntJULiP algorithm. (A) RNA-seq reads are aligned to the genome and spliced alignments are used to detect the introns and calculate their read counts. MntJULiP then tests individual introns for differential intron abundance (DSA), and groups of introns sharing a splice site ('bunches') for differential splicing ratio (DSR). (B) Left, DSA: Each intron is analyzed individually, and the expression (abundance) level is compared between conditions. Right, DSR: Introns that share a splice junction (‘bunch’) are collectively analyzed, and the PSI value for each intron is compared between conditions. Shown are: an individual exon in a three-condition experiment, in the DSA diagram, and a three-intron ‘bunch’ in a two-condition experiment, in the DSR diagram.


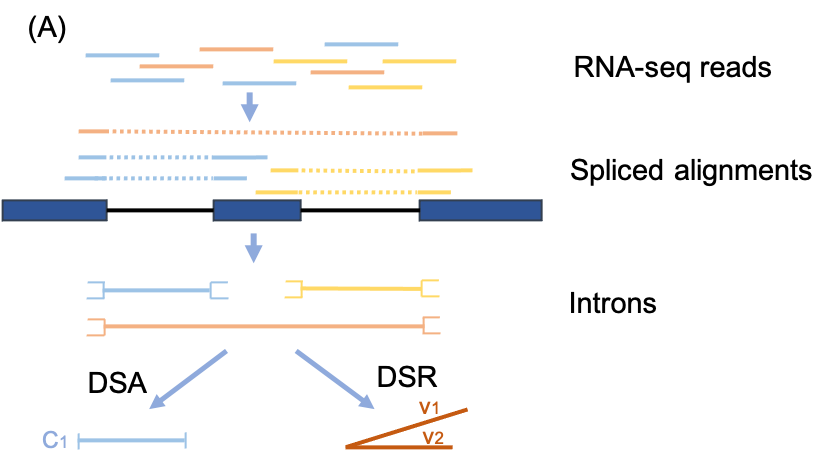

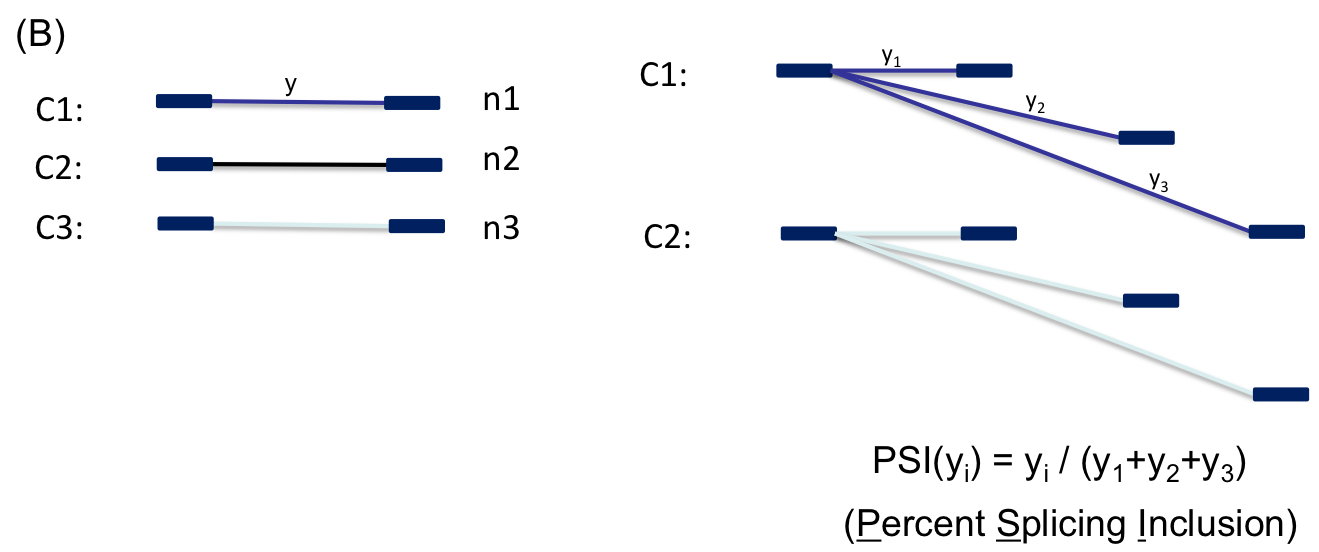

Supplement: Supplementary file 1 — Additional file 1. Supplementary figures. Supplementary Figures S1-S10. [file 13059_2022_2767_MOESM1_ESM.docx]
