# Supplementary material for: Comprehensive and scalable quantification of splicing differences with MntJULiP
Source: Genome Biol. 2022 Sep 14;23:195. doi: 10.1186/s13059-022-02767-y (PMC9472403; doi:10.1186/s13059-022-02767-y)
Supplement: Supplementary file 3 — Additional file 3. Listing of commands used in the analyses. [file 13059_2022_2767_MOESM3_ESM.docx]

DISCLAIMER: The scripts below are intended to reflect the command line parameters used with all methods and in all analyses. They have been modified from the original scripts and adapted for readability. They are NOT intended to be run ‘as it is’. The original scripts are included in the MntJULiP_scripts.tar.gz archive in the MntJULiP’s GitHub page.

**A. Simulation**

BASEDIR=/path/to/project/root/simulation3

Alignment

**STAR**

for j in ‘case’ ‘control’

do

for i in {01..25}

do

STAR --runThreadN 25 --genomeDir ${STARIDX} \

--readFilesIn ${BASEDIR}/Polyester/case/sample_${i}/sample_${i}_1.fastq.gz \

${BASEDIR}/Polyester/case/sample_${i}/sample_${i}_2.fastq.gz \

--outFileNamePrefix ${BASEDIR}/Star/case/sample_${i}/ \

--outSAMtype BAM SortedByCoordinate --outSAMstrandField intronMotif

done

done

**Hisat2**

for d in ‘case’ ‘control’

do

for i in {01..25}

do

hisat2 -p 4 -x ${H2IDX} \

-1 ${BASEDIR}/Polyester/${d}/sample_${i}/sample_${i}_1.fastq.gz \

-2 ${BASEDIR}/Polyester/${d}/sample_${i}/sample_${i}_2.fastq.gz \

| samtools view -b - \

> ${BASEDIR}/Hisat2/${d}/sample_${i}/sample_${i}.unsorted.bam

samtools sort --threads 4 -m 2G \

-o ${BASEDIR}/Hisat2/$[d}/sample_${i}/sample_${i}.bam \

${BASEDIR}/Hisat2/${d}/sample_${i}/sample_${i}.unsorted.bam

# rm ${BASEDIR}/Hisat2/${d}/sample_${i}/sample_${i}.unsorted.bam

done

done

Differential splicing:

(The Alignments directory contains a copy of the BAM files, generated with either Star or Hisat2, renamed after the sample name.)

**LeafCutter (DSR)**

(Following the instructions at: <http://davidaknowles.github.io/leafcutter/articles/Usage.html> )

bam2junc=${SWDIR}/scripts/bam2junc.sh

leafcutter_cluster=${SWDIR}/clustering/leafcutter_cluster.py

leafcutter_ds=${SWDIR}/scripts/leafcutter_ds.R

gtf_to_exons=${SWDIR}/scripts/gtf_to_exons.R

WORKDIR=${BASEDIR}/LeafCutter

for d in ‘case’ ‘control’

do

for i in {01..25}

do

sh ${bam2junc} ${BASEDIR}/Alignments/${d}/sample_${i}.bam \

${BASEDIR}/Alignments/${d}/sample_${i}.bam.junc

echo ${BASEDIR}/Alignments/${d}/sample_${i}.bam.junc >> ${WORKDIR}/test_juncfiles.txt

done

done

Rscript ${gtf_to_exons} \

${BASEDIR}/stringtie/sim.stmerged.modified_2.gtf.gz \

${BASEDIR}/meta_info/stmerged_exons.txt.gz

python ${leafcutter_cluster} -j ${WORKDIR}/test_juncfiles.txt -o ${WORKDIR}/results

for d in 'case' 'control'

do

for i in {01..25}

do

echo "${BASEDIR}/Alignments/${d}/sample_${i}.bam ${d}" >> ${WORKDIR}/test_diff_introns.txt

done

done

threads=24

${leafcutter_ds} -p ${threads} -e "${BASEDIR}/meta_info/stmerged_exons.txt.gz" \

${WORKDIR}/results_perind_numers.counts.gz \

${WORKDIR}/test_diff_introns.txt

**Filter:** p-val<0.1, |dpsi|>=0.05

**MAJIQ (DSR)**

(All computations are taking place locally, in WORKDIR. DATADIR holds a local copy of the alignment data, renamed: case_01.bam, .., case_25.bam, control_01.bam, .., control_25.bam.)

WORKDIR=${BASEDIR}/MAJIQ

cd ${BASEDIR}/MAJIQ

rm -rf config.txt

echo "[info]" >> config.txt

echo "readlen=101" >> config.txt

echo "samdir=${DATADIR}" >> config.txt

echo "genome=hg38" >> config.txt

echo "" >> config.txt

echo "[experiments]" >> config.txt

control_bams=”control_01,control_02,control_03,control_04,control_05,control_06,control_07,control_08,control_09,control_10,control_11,control_12,control_13,control_14,control_15,control_16,control_17,control_18,control_19,control_20,control_21,control_22,control_23,control_24,control_25”

case_bams=”case_01,case_02,case_03,case_04,case_05,cAse_06,case_07,case_08,case_09,case_10,case_11,case_12,case_13,case_14,case_15,case_16,case_17,case_18,case_19,case_20,case_21,case_22,case_23,case_24,case_25”

echo "CONTROL=${control_bams}" >> config.txt

echo "CASE=${case_bams}" >> config.txt

gff3=${BASEDIR}/stringtie/sim.stmerged.modified_2.gff3

majiq build ${gff3} -c config.txt -j ${threads} -o build_out

control_majiqs="build_out/control_01.majiq"

case_majiqs="build_out/case_01.majiq"

for i in {02..25}

do

control_majiqs="${control_majiqs} build_out/control_${i}.majiq"

case_majiqs="${case_majiqs} build_out/case_${i}.majiq"

done

threads=10

majiq deltapsi -grp1 `echo ${control_majiqs}` \

-grp2 `echo ${case_majiqs}` \

-j $threads \

-o dpsi_out \

-n control case

voila deltapsi dpsi_out/control_case.deltapsi.voila \

--splice-graph build_out/splicegraph.sql \

-j $threads \

--show-all \

-o voila_out

**Filter:** |dpsi|>=0.05 (also tested: p-val<0.1, |dpsi|>=0.05, which produced lower performance)

**rMATS (DSR)**

GTF=${BASEDIR}/stringtie/sim.stmerged.modified_2.gtf

control_bams="${BASEDIR}/Alignments/control/sample_01.bam"

case_bams="${BASEDIR}/Alignments/case/sample_01.bam"

for i in {02..25}

do

control_bams="${control_bams},${BASEDIR}/Alignments/control/sample_${i}.bam"

case_bams="${case_bams},${BASEDIR}/Alignments/case/sample_${i}.bam"

done

python ${SWDIR}/RNASeq-MATS.py \

-b1 ${control_bams} \

-b2 ${case_bams} \

-gtf ${GTF} \

-o rmats_out -t paired -len 101

**Filter:** p-val<0.1 (also tested: p-val<0.1, |dpsi|>=0.05, which produced lower performance)

**SUPPA2 (DSR)**

**Run salmon:**

gtf=${BASEDIR}/stringtie/sim.stmerged.modified_2.gtf

fa_file=${BASEDIR}/stringtie/sim.stmerged.modified_2.clean.fa

SIDX=${WORKDIR}/stmerged_index

DATADIR=${BASEDIR}/Polyester/

WORKDIR=${BASEDIR}/salmon

mkdir ${WORKDIR}; cd ${WORKDIR}

mkdir -p ${WORKDIR}/logs

threads=10

salmon index -p ${threads} -t ${fa_file} -i stmerged_index --gencode

for d in ‘case’ ‘control’

do

for i in {01..25}

do

mkdir -p ${WORKDIR}/${d}_sample_${i}

fastq1_prefix="${DATADIR}/${d}/sample_${i}/sample_${i}_R1"

fastq2_prefix="${DATADIR}/${d}/sample_${i}/sample_${i}_R2"

salmon quant -i $SIDX -l A -p ${core} \

-1 ${fastq1_prefix}.fastq \

-2 ${fastq2_prefix}.fastq \

-o ${WORKDIR}/${d}_sample_${i} -g ${gtf} -c 2> ${WORKDIR}/logs/${d}_sample_${i}.salmon.log

done

done

**Run SUPPA2**

WORKDIR=${BASEDIR}/suppa/

mkdir -p ${WORKDIR}; cd ${WORKDIR}

SUPPA=${SWDIR}/scripts/SUPPA/suppa.py

gtf=${BASEDIR}/stringtie/sim.stmerged.modified_2.gtf

# generate the splicing events from the annotation file

for i in SE SS MX RI FL

do

python3 ${SUPPA} generateEvents -i ${gtf} -o ${WORKDIR}/suppa -f ioe -e ${i}

done

#python3 ${SUPPA} generateEvents -i ${gtf} -o ${WORKDIR}/suppa -f ioi

mkdir -p ${WORKDIR}/tpms

for d in ‘case’ ‘control’

do

for i in {01..25}

do

python3 ${WORKDIR}/filter_tpm_4_suppa.py \

-i ${BASEDIR}/salmon/${d}_sample_${i}/quant.sf \

-o ${WORKDIR}/tpms/${d}_sample_${i}.tpm \

-s ${i}

done

done

for d in ‘case’ ‘control’

do

for i in {01..25}

do

for type in SE RI MX AL AF A5 A3

do

mkdir -p ${WORKDIR}/${type}_events/

python3 ${SUPPA} psiPerEvent -i ${WORKDIR}/suppa_${type}_strict.ioe \

-e ${WORKDIR}/tpms/${sample}.tpm \

-o ${WORKDIR}/${type}_events/${d}_sample_${i}

done

done

done

# calculate and group TPMs

cd ${WORKDIR}/tpms

for d in ‘case’ ‘control’

do

input_files=""

for i in {01..25}

do

input_files="${input_files} ${WORKDIR}/tpms/${d}_sample_${i}.tpm"

done

python3 ${SUPPA} joinFiles -i `echo ${input_files}` -f tpm -o ${d}_tpms

done

# calculate PSIs

cd ${WORKDIR}/tpms

input_files=""

d='control'

for type in SE RI MX AL AF A5 A3

do

cd ${WORKDIR}/${type}_events

for i in {01..25}

do

input_files="${input_files} ${WORKDIR}/${type}_events/${d}_sample_${i}.psi"

done

python3 ${SUPPA} joinFiles -i `echo ${input_files}` \

-f psi \

-o ${d}_psis

done

# classical (sufficient # of samples) versus empirical

for type in SE RI MX AL AF A5 A3

do

for method in classical empirical

do

cd ${WORKDIR}/${type}_events

python3 ${SUPPA} diffSplice --method ${method} \

--input ${WORKDIR}/suppa_${type}_strict.ioe \

--psi ${WORKDIR}/${type}_events/control_psis.psi \

${WORKDIR}/${type}_events/case_psis.psi \

--tpm ${WORKDIR}/tpms/control_tpms.tpm ${WORKDIR}/tpms/case_tpms.tpm \

-gc \

-o ${type}_${method}_dpsis

done

done

**Filter:** p-val<0.1 (also tested: p-val<0.1, |dpsi|>=0.05, which produced lower performance)

**MntJULiP (DSR and DSA)**

WORKDIR=${BASEDIR}/MntJULiP

MNTJULIP=${SWDIR}/MntJULiP-master/mntjulip

rm -rf sim3.bamlist.txt

echo “sample\tcondition” >> sim3.bamlist.txt

for d in ‘control’ ‘case’

do

for i in {01..25}

do

echo “${BASEDIR}/Alignments/${d}/sample_${i}.bam ${d}” >> sim3.bamlist.txt

done

done

threads=10

${MNTJULIP} --out-dir ${WORKDIR} \

--bam-list ${WORKDIR}/sim3.bamlist.txt \

--num-threads ${threads} \

--batch-size 2000 \

--min-count 1 \

--anno-file ${BASEDIR}/stringtie/sim.stmerged.modified_2.gtf

**Filter:** p-val<0.1, |dpsi|>=0.05 (DSR) ;

p-val<0.1 (DSA)

**Cuffdiff2 (DSA)**

threads=10

WORKDIR=${BASEDIR}/Cuffdiff

GTF=${BASEDIR}/stringtie/sim.stmerged.modified_2.gtf

control_bams=”${BASEDIR}/Alignments/control/sample_01.bam”

case_bams="${BASEDIR}/Alignments/case/sample_01.bam”

for i in {02..25}

do

control_bams=${control_bams},${BASEDIR}/Alignments/control/sample_${i}.bam

case_bams=${case_bams},${BASEDIR}/Alignments/case/sample_${i}.bam

done

cuffdiff -o ${WORKDIR} -p threads ${GTF} ${ctrl_samples} ${case_samples}

**Filter:** p-val<0.1

**SLEUTH (DSA)**

**Run Kallisto:**

DATADIR=${BASEDIR}/Polyester/

WORKDIR=${BASEDIR}/kallisto

gtf=${BASEDIR}/stringtie/sim.stmerged.modified_2.gtf

fa_file=${BASEDIR}/stringtie/sim.stmerged.modified_2.clean.fa # ‘exon’ lines only

kallisto index -i index ${fa_file}

mkdir -p ${WORKDIR}; cd ${WORKDIR}

threads=10

for d in ‘case’ ‘control’

do

for i in {01..25}

do

fastq1="${DATADIR}/${d}/sample_${i}/sample_${i}_R1.fastq"

fastq2="${DATADIR}/${d}/sample_${i}/sample_${i}_R2.fastq"

kallisto quant -i index -o ${d}_sample_${i} -b 10 \

-t ${threads} -g ${gtf} \

${fastq1} ${fastq2}

done

done

echo "sample\tcondition\tpath" > metadata.txt

for d in ‘case’ ‘control’

do

for i in {01..25}

do

echo "${d}_sample_${i}\t${d}\t${WORKDIR}/${d}_sample_${i}\n" >> metadata.txt

done

done

**Sleuth (in R):**

file <- “metadata.txt"

s2c <- read.table(file, header=TRUE, stringsAsFactors=FALSE)

# colnames(s2c)

custom_filter <- function(row, min_reads=1, min_prop=0.001) {

mean(row >= min_reads) >= min_prop

}

so <- sleuth_prep(s2c, num_cores=10, filter_fun=custom_filter)

so <- sleuth_fit(so, ~condition, 'full')

so <- sleuth_fit(so, ~1, 'reduced')

so <- sleuth_lrt(so, 'reduced', 'full')

sleuth_table <- sleuth_results(so, 'reduced:full', 'lrt', show_all = FALSE)

# sleuth_significant <- dplyr::filter(sleuth_table, qval <= 0.05)

head(sleuth_table, 20)

file <- ./kallisto_out.csv"

ret <- write.csv(x=sleuth_table, file=file)

**Filter:** p-val<0.1

**B. Hippocampus**

BASEDIR=/path/to/project/root/Hippocampus

sample_list= “ERR1779513 ERR1779503 ERR1779502 ERR1779500 ERR1779491 ERR1779489 ERR1779487 ERR1779457 ERR1779452 ERR1779451 ERR1779449 ERR1779444 ERR1779443 ERR1779435 ERR1779432 ERR1779430 ERR1779422 ERR1779420 ERR1779417 ERR1779414 ERR1779382 ERR1779379 ERR1779372 ERR1779370 ERR1779367 ERR1779363 ERR1779355 ERR1779353 ERR1779351 ERR1779350 ERR1779349 ERR1779346 ERR1779344 ERR1779343 ERR1779334 ERR1779333 ERR1779332 ERR1779327 ERR1779321 ERR1779320 ERR1779317 ERR1779309 ERR1779301 ERR1779300”

control_list=” ERR1779355 ERR1779332 ERR1779372 ERR1779317 ERR1779333 ERR1779382 ERR1779363 ERR1779327 ERR1779353 ERR1779300 ERR1779349 ERR1779367 ERR1779344 ERR1779346 ERR1779350 ERR1779334 ERR1779379 ERR1779320 ERR1779309 ERR1779321 ERR1779370 ERR1779351 ERR1779301 ERR1779343”

tail_control_list=” ERR1779332 ERR1779372 ERR1779317 ERR1779333 ERR1779382 ERR1779363 ERR1779327 ERR1779353 ERR1779300 ERR1779349 ERR1779367 ERR1779344 ERR1779346 ERR1779350 ERR1779334 ERR1779379 ERR1779320 ERR1779309 ERR1779321 ERR1779370 ERR1779351 ERR1779301 ERR1779343”

epileptic_list=” ERR1779449 ERR1779513 ERR1779451 ERR1779414 ERR1779444 ERR1779452 ERR1779491 ERR1779420 ERR1779435 ERR1779430 ERR1779502 ERR1779500 ERR1779457 ERR1779432 ERR1779487 ERR1779489 ERR1779417 ERR1779443 ERR1779503 ERR1779422”

tail_epileptic_list=” ERR1779513 ERR1779451 ERR1779414 ERR1779444 ERR1779452 ERR1779491 ERR1779420 ERR1779435 ERR1779430 ERR1779502 ERR1779500 ERR1779457 ERR1779432 ERR1779487 ERR1779489 ERR1779417 ERR1779443 ERR1779503 ERR1779422”

Alignment:

**STAR**

(Example shown for one sample, ERR1779300)

for i in ${sample_list}

do

star --runThreadN 20 --genomeDir /${STARIDX} \

--readFilesIn ${BASEDIR}/data/${i}_1.fastq.gz \

${BASEDIR}/data/${i}_2.fastq.gz \

--readFilesCommand zcat \

--limitBAMsortRAM 10000000000 \

--outFileNamePrefix ${BASEDIR}/Star/${i}/ \

--outSAMtype BAM SortedByCoordinate --outSAMstrandField intronMotif

done

**MntJULiP (DSR and DSA)**

WORKDIR=${BASEDIR}/MntJULiP/

mkdir -p ${WORKDIR} ; cd ${WORKDIR}

#The ‘hippocampus.bam.txt’ file is a 2-column TSV file that lists the BAM files and associated condition, for example:

**#**sample condition

#/scratch/gyang/Hippocampus/Star/ERR1779300/Aligned.sortedByCoord.out.bam control

#/scratch/gyang/Hippocampus/Star/ERR1779349/Aligned.sortedByCoord.out.bam control

#...

echo -e "sample\tcondition" > ${WORKDIR}/hippocampus.bam.txt

for i in ${control_list}

do

echo -e "${BASEDIR}/Star/${i}/Aligned.sortedByCoord.out.bam\tcontrol" >> ${WORKDIR}/hippocampus.bam.txt

done

for i in ${epileptic_list}

do

echo -e "${BASEDIR}/Star/${i}/Aligned.sortedByCoord.out.bam\tepileptic" >> ${WORKDIR}/hippocampus.bam.txt

done

threads=10

mntjulip --out-dir ${WORKDIR} \

--bam-list ${WORKDIR}/hippocampus.bam.txt \

--num-threads ${threads} \

--min-count 5 \

--batch-size 2000 \

--anno-file ${BASEDIR}/gencode.vM17.annotation.gtf

**Filter:** p-val<0.0.5, |dpsi|>=0.05 (DSR)

P-val<0.05 (DSA)

**LeafCutter**

#NOTE: DATADIR holds a copy of the alignment data, renamed: ERR1779300.bam, etc.

DATADIR=${BASEDIR}/data

### Generate the required ‘test_juncfiles.txt’

for d in ${sample_list}

do

sh ${bam2junc} ${DATADIR}/${d}.bam ${DATADIR}/${d}.bam.junc

echo ${DATADIR}/${d}.bam.junc >> test_juncfiles.txt

done

mkdir results; cd results

python ${leafcutter_cluster} -j ../test_juncfiles.txt -o results

### generate exon file

Rscript ${SWDIR}/scripts_to_exons.R ${BASEDIR}/gencode.vM17.annotation.gtf.gz ${WORKDIR}/exons.txt.gz

### generate the test_diff_introns.txt file

t='control'

for d in ${control_list}

do

echo "${d}.bam ${t}" >> test_diff_introns.txt

done

t='epileptic'

for d in ${epileptic_list}

do

echo "${d}.bam ${t}" >> test_diff_introns.txt

done

threads=10

${leafcutter_ds} -p ${threads} -e "${WORKDIR}/exons.txt.gz" \

results_perind_numers.counts.gz \

test_diff_introns.txt

**Filter:** p-val<0.05, |dpsi|>=0.05

**MAJIQ**

WORKDIR=${BASEDIR}/MAJIQ

mkdir -p ${WORKDIR}/results ; cd ${WORKDIR}/results

rm -rf config.txt

echo "[info]" >> config.txt

echo "readlen=75" >> config.txt

echo "samdir=${LOCAL_DATA_DIR}" >> config.txt

echo "genome=mm10" >> config.txt

echo "" >> config.txt

echo "[experiments]" >> config.txt

control_bams=”ERR1779355,ERR1779332,ERR1779372,ERR1779317,ERR1779333, ERR1779382,ERR1779363,ERR1779327,ERR1779353,ERR1779300,ERR1779349, ERR1779367,ERR1779344,ERR1779346,ERR1779350,ERR1779334.ERR1779379, ERR1779320,ERR1779309,ERR1779321,ERR1779370,ERR1779351,ERR1779301, ERR1779343”

epileptic_bams="ERR1779449,ERR1779513,ERR1779451 ERR1779414,

ERR1779444,ERR1779452,ERR1779491,ERR1779420,ERR1779435,ERR1779430, ERR1779502,ERR1779500,ERR1779457,ERR1779432,ERR1779487,ERR1779489, ERR1779417,ERR1779443,ERR1779503,ERR1779422”

echo "CONTROL=${control_bams}" >> config.txt

echo "EPILEPTIC=${epileptic_bams}" >> config.txt

gff3=${BASEDIR}/gencode.vM17.annotation.gff3

threads=10

majiq build ${gff3} -c config.txt -j $threads -o build_out

control_majiqs="build_out/ERR1779355.majiq"

for d in ${tail_control_list}

do

control_majiqs="${control_majiqs} build_out/${d}.majiq"

done

epileptic_majiqs="build_out/ERR1779449.majiq"

for d in ${tail_epileptic_list}

do

epileptic_majiqs="${epileptic_majiqs} build_out/${d}.majiq"

done

threads=10

majiq deltapsi -grp1 `echo ${control_majiqs}` \

-grp2 `echo ${epileptic_majiqs}` \

-j $threads \

-o dpsi_out \

-n control epileptic

voila deltapsi dpsi_out/control_epileptic.deltapsi.voila \

--splice-graph build_out/splicegraph.sql \

-j $threads \

--show-all \

-o voila_out

**Filter:** |dpsi|>=0.05

**rMATS:**

control_rmats_bams="${BASEDIR}/Star/ERR1779355/Aligned.sortedByCoord.out.bam"

for i in ${tail_control_list}

do control_rmats_bams="${control_rmats_bams},${BASEDIR}/Star/${i}/Aligned.sortedByCoord.out.bam"

done

epileptic_rmats_bams="${BASEDIR}/Star/ERR1779449/Aligned.sortedByCoord.out.bam"

for i in ${tail_epileptic_list}

do

epileptic_rmats_bams="${epileptic_rmats_bams},${BASEDIR}/Star/${i}/Aligned.sortedByCoord.out.bam"

done

python ${RMATS} \

-b1 ${control_rmats_bams} \

-b2 ${epileptic_rmats_bams} \

-o out -t paired -len 75

**Filter:** p-val<0.05, |dpsi|>=0.05

**SUPPA2**

**Run salmon:**

WORKDIR=${BASEDIR}/salmon

DATADIR=${BASEDIR}/data # contains the FASTQ sequences

gtf=${BASEDIR}/gencode.vM17.annotation.gtf

fa_file=${BASEDIR}/ gencode.vM17.annotation.fa

SIDX=${WORKDIR}/transcripts_index

mkdir ${WORKDIR}; cd ${WORKDIR}

threads=10

salmon index -p ${threads} -t ${fa_file} -i transcripts_index --gencode

mkdir -p ${WORKDIR}/logs

for i in ${sample_list}

do

mkdir -p ${WORKDIR}/${i}

salmon quant -i $SIDX -l A -p ${threads} \

-1 ${DATADIR}/${i}_1.fastq.gz \

-2 ${DATADIR}/${i}_2.fastq.gz \

-o ${WORKDIR}/${i} -g ${gtf} -c 2\

> ${WORKDIR}/logs/${i}.salmon.log

done

**Run SUPPA:**

WORKDIR=${BASEDIR}/suppa

SUPPA=${SWDIR}/ SUPPA/suppa.py

gtf=${BASEDIR}/gencode.vM17.annotation.gtf

mkdir -p ${workdir}; cd ${workdir}

# generate the splicing events from the annotation file

python3 ${SUPPA} generateEvents -i ${gtf} -o ${WORKDIR}/suppa -f ioe -e SE &

python3 ${SUPPA} generateEvents -i ${gtf} -o ${ WORKDIR}/suppa -f ioe -e SS &

python3 ${SUPPA} generateEvents -i ${gtf} -o ${ WORKDIR}/suppa -f ioe -e MX &

python3 ${SUPPA} generateEvents -i ${gtf} -o ${ WORKDIR}/suppa -f ioe -e RI &

python3 ${SUPPA} generateEvents -i ${gtf} -o ${WORKDIR}/suppa -f ioe -e FL &

mkdir -p ${WORKDIR}/tpms

for i in ${sample_list}

do

python3 ${WORKDIR}/filter_tpm_4_suppa.py \

-i ${BASEDIR}/salmon/${i}/quant.sf \

-o ${WORKDIR}/tpms/${i}.tpm \

-s ${i}

done

n=24

k=0

for i in ${sample_list}

do

for type in SE RI MX AL AF A5 A3

do

mkdir -p ${WORKDIR}/${type}_events/

python3 ${SUPPA} psiPerEvent -i ${WORKDIR}/suppa_${type}_strict.ioe \

-e ${WORKDIR}/tpms/${i}.tpm \

-o ${WORKDIR}/${type}_events/${i} &

k=$(($k+1))

if [ $k -eq $n ]; then

wait

k=0

fi

done

done

python3 ${SUPPA} generateEvents -i ${gtf} -o ${WORKDIR}/suppa -f ioi

cd ${WORKDIR}/tpms

input_files=""

d='control'

for i in ${control_list}

do

input_files="${input_files} ${WORKDIR/tpms/${i}.tpm"

done

python3 ${SUPPA} joinFiles -i `echo ${input_files}` -f tpm -o ${d}_tpms

input_files=""

d='epileptic'

for i in ${epileptic_list}

do

input_files="${input_files} ${WORKDIR}/tpms/${i}.tpm"

done

python3 ${SUPPA} joinFiles -i `echo ${input_files}` -f tpm -o ${d}_tpms

cd ${WORKDIR}/tpms

input_files=""

d='control'

for type in SE RI MX AL AF A5 A3

do

cd ${WORKDIR/ ${type}_events

for i in ${control_list}

do

input_files="${input_files} ${WORKDIR}/${type}_events/${i}.psi"

done

python3 ${SUPPA} joinFiles -i `echo ${input_files}` -f psi -o ${d}_psis

done

input_files=""

d='epileptic'

for type in SE RI MX AL AF A5 A3

do

cd ${WORKDIR}/${type}_events

for i in ${epileptic_list}

do

input_files="${input_files} ${WORKDIR}/ ${type}_events/${i}.psi"

done

python3 ${SUPPA} joinFiles -i `echo ${input_files}` -f psi -o ${d}_psis

done

for type in SE RI MX AL AF A5 A3

do

for method in ‘classical’ ‘empirical’

do

cd ${WORKDIR}/ ${type}_events

python3 ${SUPPA} diffSplice --method ${method} \

--input ${WORKDIR}/suppa_${type}_strict.ioe \

--psi ${WORKDIR}/${type}_events/control_psis.psi ${WORKDIR}/${type}_events/epileptic_psis.psi \

--tpm ${WORKDIR}/tpms/control_tpms.tpm ${WORKDIR}/ tpms/epileptic_tpms.tpm \

-gc \

-o ${type}_${method}_dpsis

done

done

**Filter:** p-val<0.05, |dpsi|>=0.05

**C. GTEx (Frontal cortex, Cortex, Cerebellum, Lung)**

BASEDIR=/path/to/project/root/GTEx

**STAR**

for i in `cat sample_list.txt`

do

STAR --runThreadN 8 \

--genomeDir $STAR2IDX/ \

--readFilesIn ${DATADIR}/${i}_1.fastq.gz ${DATADIR}/${i}_2.fastq.gz \

--readFilesCommand zcat \

--outSAMtype BAM SortedByCoordinate \

--outSAMstrandField intronMotif \

--outFileNamePrefix star_${i}_

done

**MntJULiP**

**Run Junc** (create splice files)

(Example shown for Cerebellum; ${WORKDIR}/Cerebellum/Alignments/ contains a copy of the STAR BAM files for all cerebellum samples, renamed after the sample id, e.g. ERR123456.)

mkdir ${BASEDIR}/Cerebellum/SPLICE

for i in `cat cerebellum_sample_list.txt`

do

junc ${BASEDIR}/Cerebellum/Alignments/${i} -a > ${BASEDIR}/Cerebellum/SPLICE/${i}.splice

done

**Run MntJULiP**

*Pairwise comparisons:*

## Example: cortex vs cerebellum

WORKDIR=${BASEDIR}/cortex_cerebellum_out

mntjulip --out-dir ${WORKDIR} \

--splice-list ${BASEDIR}/cortex_cerebellum.splice.txt \

--num-threads 23 \

--min-count 5\

--batch-size 2000 \

--anno-file ${BASEDIR}/gencode.v22.annotation.gtf

Where ‘cortex_cerebellum.splice.txt’ is a .txt file with two columns separated by ‘\t’, listing the per sample splice file and sample group, for example:

sample condition

path_to_sample1.splice Cerebellum

path_to_sample2.splice Cortex

The same commands apply to all pairwise comparisons: cortex_frontalcortex, cortex_lung, frontalcortex_cerebellum, cerebellum-lung.

**Filter (for visualizations):** p-val<0.05, |dpsi|>=0.2 (DSR) ; p-val<0.05 (DSA)

*Multi-way comparisons:*

## Example: cortex_cerebellum_lung

WORKDIR="${BASEDIR}/cortex_cerebellum_lung_out"

mntjulip --out-dir ${WORKDIR} \

--splice-list ${BASEDIR}/GTEx.cortex_cerebellum_lung.splice.txt \

--num-threads 23 \

--min-count 5\

--batch-size 2000 \

--anno-file ${BASE_DIR}/gencode.v22.annotation.gtf

**Filter:** p-val<0.05, |dpsi|>=0.2 (DSR) ; p-val<0.05 (DSA)

**D. Organoid (**DRA005238)

BASEDIR=/path/to/project/root/DRA005238

organoid_sample_list=”DRR075852 DRR075854 DRR075856 DRR075858 DRR075860 DRR075862 DRR075864 DRR075853 DRR075855 DRR075857 DRR075859 DRR075861 DRR075863 DRR075865”

**STAR**

for i in ${organoid_sample_list}

do

STAR --runThreadN 8 \

--genomeDir ${STAR2IDX} \

--readFilesIn ${BASEDIR}/Data/${i}.fastq.bz2 \

--readFilesCommand bzcat \

--outSAMtype BAM SortedByCoordinate \

--outSAMstrandField intronMotif \

--outFileNamePrefix star_${name}_

done

**MntJULiP:**

*Pairwise comparisons (all-against-all):*

for dir in all 2D_4D 2D_6D 2D_8D 2D_10D 2D_12D 2D_14D 4D_6D 4D_8D 4D_10D 4D_12D 4D_14D 6D_8D 6D_10D 6D_12D 6D_14D 8D_10D 8D_12D 8D_14D 10D_12D 10D_14D 12D_14D

do

WORKDIR=${BASEDIR}/${dir}

mntjulip --out-dir ${WORKDIR} \

--bam-list ${WORKDIR}/bam_list.txt \

--num-threads 10 \

--min-count 20 \

--group-filter 15 \

--batch-size 2000 \

--anno-file ${BASEDIR}/gencode.vM17.annotation.gtf

done

*Multi(7)-way comparison:*

WORKDIR=${BASEDIR}/all

mntjulip --out-dir ${WORKDIR} \

--bam-list ${WORKDIR}/bam_list.txt \

--num-threads 10 \

--min-count 20 \

--batch-size 2000 \

--group-filter 15 \

--anno-file ${BASEDIR}/gencode.vM17.annotation.gtf

**Filter:** p-val<0.05, |dpsi|>=0.05 (DSR) ; p-val<0.05 (DSA)

**Leafcutter:**

(LOCAL_DATADIR contains links to sample alignment files, renamed after the sample name, e.g. DRR075856.bam.)

LOCAL_DATADIR=${BASEDIR}/LeafCutter/data

bam2junc=${SWDIR}/scripts/bam2junc.sh

leafcutter_cluster=${SWDIR}/clustering/leafcutter_cluster.py

leafcutter_ds=${SWDIR}/scripts/leafcutter_ds.R

gtf_to_exons=${SWDIR}/scripts/gtf_to_exons.R

for i in ${organoid_sample_list}

do

sh ${bam2junc} ${LOCAL_DATADIR}/${i}.bam ${LOCAL_DATADIR}/${i}.bam.junc

echo ${LOCAL_DATA_DIR}/${i}.bam.junc >> test_juncfiles.txt

done

Rscript ${gtf_to_exons} ${BASEDIR}/gencode.vM17.annotation.gtf.gz ${LOCAL_DATADIR}/exons.txt.gz

## 2D vs 4D (and similarly for the other comparisons)

WORKDIR=${BASEDIR}/LeafCutter/2D_4D

mkdir -p ${WORKDIR} ; cd ${WORKDIR}

for name in DRR075852 DRR075859 DRR075853 DRR075860

do

echo ${LOCAL_DATA_DIR}/${name}.bam.junc >> test_juncfiles.txt

done

python ${leafcutter_cluster} -j test_juncfiles.txt -o results

for name in DRR075852 DRR075859

do

echo "${name}.bam 2D" >> test_diff_introns.txt

done

for name in DRR075853 DRR075860

do

echo "${name}.bam 4D" >> test_diff_introns.txt

done

threads=10

${leafcutter_ds} -i 2 -g 2 -p ${threads} \

-e "${LOCAL_DATADIR}/exons.txt.gz" \

results_perind_numers.counts.gz \

test_diff_introns.txt

**Filter:** p-val<0.05, |dpsi|>=0.05

**GTEX_brain:**

BASEDIR=/path/to/project/root/GTEx_brain

WORKDIR=${BASEDIR}/MntJulip_out

*(All) Pairwise comparisons:*

for comparison in `cat comparison_list.txt`

do

out_dir=${WORKDIR}/${comparison}

mntjulip --out-dir ${out_dir} \

--splice-list ${out_dir}/splice_list.txt \

--num-threads 24 \

--min-count 5 \

--batch-size 2000 \

--anno-file ${BASEDIR}/gencode.v36.annotation.gtf

done

*Multi(13)-way comparison:*

out_dir=${WORKDIR}/13_brain_tissues

mntjulip --out-dir ${out_dir} \

--splice-list ${out_dir}/splice_list.txt \

--num-threads 24 \

--min-count 5 \

--batch-size 2000 \

--anno-file ${BASEDIR}/gencode.v36.annotation.gtf

**Filter:** p-val<0.05, |dpsi|>=0.05
