# Supplementary material for: Comprehensive and scalable quantification of splicing differences with MntJULiP
Source: Genome Biol. 2022 Sep 14;23:195. doi: 10.1186/s13059-022-02767-y (PMC9472403; doi:10.1186/s13059-022-02767-y)
Supplement: Supplementary file 4 — Additional file 4. Peer review history. [file 13059_2022_2767_MOESM4_ESM.docx]

**Review history**

**First round of review**

**Reviewer 1**

The authors describe a novel algorithm to detect and find differential splicing events. The algorithm is based on a bayesian analysis using a zero-inflated negative binomial distribution whose parameters are identified by maximum likelihood estimation.
The authors discern two different statistical tests: DSA and DSR. The first one tests for the difference of expression of the event under consideration and the second tests for the difference of relative expression. Both tests have their own scope and the authors illustrate their imporatance by performing a GO enrichment analysis and showing the differences in the significant terms.
The algorithm is compared against Majiq, rMats, Leafcutter and CuffDiff2 using simulated and real datasets.
Validation of splicing events in the case of real data is always a challenge and the authors use as a proxy, how coherent are the results across different algorithms. Assuming that an event identified by several algorithms is a "true positive" (kindof), and that events identified by only one method are likely "false positives", they state that, for a similar number of discoveris, their method have the lowest false discovery rate.

Comments:
Major.
There are methods that do not find novel events such as SUPPA2 are are not included in the comparison. In addition, Sleuth could also be included in the DSA analysis since it includes an interesting approach based on bootstrapping to find differentially expressed isoforms.

As long as I understood, this method only uses the junction reads (maybe I am wrong) and exon reads are not included in the study. From my point of view, this approach is not optimal and strongly depends on the ability of STAR or HISAT2 to map junction reads. It would also be interesting if the authors found differences between both mapping algorithms and state in the main manuscript or the additional material the recommended parameters to perform the mapping.

It would be interesting to include a profile of time and memory require to perform the whole analysis and compare this time with other methods. Harware characteristics (number of cores, processor type and so on) should also be included.

The proposed algorithm does not classify the event according to the "canonical" categories (cassette exon, intron retention, alternative 3', alternative 5', mutually exclusive exons). Is it possible to identify the splicing event?

One of the difficulties that I found using MAJIQ is that a single splicing event (a cassette exon for instance) is represented by two different statistics. Does this algorithm integrate both pieces of information into a single number?

Matching the splicing events from different tools is not trivial at all. For example, rMAts and MAJIQ have a very different definition of splicing event difficult to match. Could the authors state how they did it?

Minor:
In the zero inflated NB, pi and mu are related to each other. If I am right, the chi-2 statistical test that compares log-likelihoods will be significant if any of the paremeters of the NB changes (either mu, sigma or pi). Perhaps, events with low.pvalues are not too interesting (for example a change in pi or in the variance). This consideration does not invalidate the analysis (in fact this is a minor comment) but shows the need to set a threshold in PSI.

One advantage of MntJULiP is the possibility to study several conditions at once. Is there any possibility to include also a continuous factor instead of an indicial one? Let me illustrate this with an example: would it be possible to find splicing events that are correlated with the body mass index for example? In other words, can a general design matrix be used with MntJULiP?

For a more clear representation, the Venn diagrams could be proportional or using an Upset plot. There are some packages in R (and probably also in Python) easy to use, customizable and with a very good output for both upset and propotional Venn diagrams.

There are wrong characters (question marks) in Figure 1C.

Figure 1D. Perhaps the rows could be ordered using a tree to show more apparently the differences between the samples.

**Reviewer 2**

The paper proposed an algorithm called MntJULip to detect differentially expressed alternative splicing events from both pair-wise comparison and multi-group comparison. The algorithm can do both DSA and DSR test. Thus it offers a one-size-fits-all approach that is not commonly found in other existing algorithms. The paper has conducted a variety of experiments using several different datasets and reported outstanding performance of MntJUlip in comparison with other existing algorithms. However, more details and explanations are needed to understand the novelty and robustness of the proposed approach.

1. The metric for comparison between MntJULip and Cuffdiff2 is not quite clear as MntJulip is trying to quantify intron while cuffdiff2 quantifies isoform abundance.

2. As with most of the alternative splicing analysis algorithms, the program also consists of two parts, builder and quantifier. The first step basically extracts all the introns while the second step conducts a statistical test on these introns. It would be revealing to see which step of the two contributes to the high sensitivity and high precision of the algorithm in comparison to other algorithms for both DSA and DSR detection.

3. In the simulated dataset, the authors claim that the high false-positive rates for MAJIQ, rMATs and Cuffdif2 were due to genes outside the simulated gene set. This in fact is a difficult problem. However, it is unclear from the method description how the MntJulip can address this specific challenge.

It is also unclear why the transcript assembly from string-tie were fed to other algorithms for this experiment. String-tie itself might contain false-positive results thus affecting the accuracy of the algorithms relying on it as reference.

4. The authors claimed MntJUlip may detect a variety of alternative splicing events. But the algorithm is indeed intron centric. It only tells which intron is differentially expressed but really cannot tell what alternative splicing is going on. It will need a great deal of digging and interpretation to get a complete picture of alternative splicing events.

5. There does not exist detailed description of different parameters used for other algorithms. It is not clear if the optimal setting for other algorithms has been attempted. For example, MntJULip used a threshold of 3 to filter introns. Did other algorithms also take similar threshold? Such threshold could be crucial for performance difference.

6. There are certain parameters required to be set within MntJUlip's statistical model. It is unclear how it was set in each experiment. It is interesting to see the performance as a function of different parameters including the minimum read count used to filter lowly expressed introns.

7. The figures reporting the performance comparison should only include algorithms in the same category, i.e., either DSA related algorithm or DSR related algorithm.

**Authors’ response to reviewers**

We wish to thank the Editor and the two referees for their careful and thoughtful evaluation of our manuscript and constructive remarks. We revised our manuscript to address their comments and concerns, providing additional explanations and analyses to demonstrate our program’s robustness and high performance, and the advance it represents over other methods. In particular, we added two reference tools, SUPPA2 and Sleuth, to our comparative evaluation; evaluated all programs with an alternate set of alignments generated by Hisat2; provided runtime and memory usage information for all programs; compiled a list of ‘recipes’ (commands) used for each program and in each experiment; undertook an analysis showing the effects of the two algorithmic components (‘builder ‘and ‘quantifier’) on the programs’ performance; and conducted an analysis showing the robustness of MntJULiP with user-adjustable parameters. Additionally, we provided explanations regarding the outcomes, interpretations, and possible extensions of the program, and updated and/or improved several of the figures. For easy reference, we provided the document with the marked Tracked Changes in addition to the final revised document, and a point-by-point response to the reviewers’ questions is enclosed below.

**Reviewer #1:**

Q1. “Major.
There are methods that do not find novel events such as SUPPA2 are not included in the comparison. In addition, Sleuth could also be included in the DSA analysis since it includes an interesting approach based on bootstrapping to find differentially expressed isoforms.”

*Answer:*

We thank the reviewer for bringing these two tools to our attention. Of note, the two methods also distinguish themselves as a class as they do not use genomically aligned reads; rather, they use pseudoalignment to obtain a fast estimate of transcript expression levels, which are then used in quantification. We added the two methods to the list of tools presented in the simulation and the mouse hippocampus experiments, and their results were incorporated in a revised **Figure 1** (**A** and **B**)**,** and in **Supplementary Figure S1**. We find that MntJULiP maintains its advantage for both the DSR and DSA formulations. Additionally, SUPPA2 shows low sensitivity likely due to its analytical method being less sensitive in the low-variance controlled simulation environment as well as to the large database size and reliance on potentially inaccurate transcript expression estimates, and Sleuth has high sensitivity but low precision due to false positives potentially caused by inaccuracies in the transcript set and transcript expression levels.

Q2. “As long as I understood, this method only uses the junction reads (maybe I am wrong) and exon reads are not included in the study. From my point of view, this approach is not optimal and strongly depends on the ability of STAR or HISAT2 to map junction reads. It would also be interesting if the authors found differences between both mapping algorithms and state in the main manuscript or the additional material the recommended parameters to perform the mapping.”

*Answer:*

We appreciate the reviewer’s insights. While, intuitively, using additional evidence can lead to improved results, the performance of any method depends on multiple factors. We point out that LeafCutter, a popular software that we include in our analysis, also uses only splice junction reads.

With regards to the alignment software, while MntJULiP was developed using alignments produced by STAR and Tophat2, we tested and now include an evaluation of all methods based on Hisat2 alignments. Program performance on the two alignment sets was comparable for most methods, with MntJULiP showing a slight decrease and LeafCutter a small increase in precision, while rMATS and Cuffdiff suffered a larger decrease in overall performance. Results for Sleuth and SUPPA2 remained unchanged, since they did not use genomic alignments.

Lastly, we included as ‘**Additional File 3’** a document providing the parameters used for alignment and all differential splicing tools, and the original scripts used in all of our analyses are included with the GitHub project.

Q3. “It would be interesting to include a profile of time and memory require to perform the whole analysis and compare this time with other methods. Hardware characteristics (number of cores, processor type and so on) should also be included.”

*Answer:*

**Supplemental Figure 1C** now shows the estimated runtimes, both wall clock and total, or ‘sequential’, and memory requirements for all programs on the simulated data, and included a discussion in the manuscript in the section “**Performance evaluation on the simulated data”**. We found that MntJULiP, LeafCutter, SUPPA2 and Sleuth had runtimes within a close range (e.g., 4-20 min wall clock time), whereas rMATS and Cuffdiff were significantly slower. Further, programs were relatively close in terms of memory requirements. All measurements were performed on Hisat2 alignments, where applicable. MAJIQ could not be tested since the original architecture was migrated and MAJIQ relies on libraries that have recently become proprietary.

Q4. “The proposed algorithm does not classify the event according to the "canonical" categories (cassette exon, intron retention, alternative 3', alternative 5', mutually exclusive exons). Is it possible to identify the splicing event?”

*Answer:*

No, it is not possible to further classify the event without performing an assembly operation to reconstruct the full-length transcripts, which would introduce inaccuracies and is antithetic to the principles on which MntJULiP was based, namely assembly-free discovery of gene differential splicing. Additionally, some alternative splicing patterns (see examples in **Supplementary Figure S5**) cannot be expressed in the framework of the primitive event classes. Note that other tools that focus on primitive types of events (exon, introns), such as DEXseq or LeafCutter, also do not pinpoint the type of event.

Q5. “One of the difficulties that I found using MAJIQ is that a single splicing event (a cassette exon for instance) is represented by two different statistics. Does this algorithm integrate both pieces of information into a single number?”

*Answer:*

We thank the reviewer for the keen observation. No, they are not integrated. Other representation drawbacks exist. For instance, by the way introns are grouped (i.e., a ‘bunch’ represents a group of introns that share a splice junction) the program may report an intron, for instance the spanning intron in a skipped exon event, in two different ‘bunches’. Also, two or more introns may be reported from the same ‘bunch’. While MntJULiP makes no effort to resolve these, our visualization software Jutils uses a procedure to select an unbiased set of introns for visualization. We included a link to the method description in the GitHUb README.md document for MntJULiP, which the users can use as a model to filter the results of their analyses.

Q6. “Matching the splicing events from different tools is not trivial at all. For example, rMAts and MAJIQ have a very different definition of splicing event difficult to match. Could the authors state how they did it?”

*Answer:*

This is indeed a very difficult operation and, in fact, one that is not always possible. As it was noted, different methods use different criteria, which may point to different types of features (introns, exons, events) exhibiting differential splicing. Oftentimes these features represent distinct splicing patterns occurring at different locations within the gene, which makes them impossible to relate in the absence of a reference set of gene and transcript annotations, as is the case for instance for MntJULiP and LeafCutter.

In our analysis we simulated differential splicing broadly, by swapping the expression levels of the top two isoforms regardless of their splicing patterns, and used the gene as the unit of differential splicing information. Hence, for each program tested we consider the list of genes for which the program reports differentially spliced features, and compare it to the set of simulated genes. For clarity, we included the following explanation in the manuscript: *“Different methods employ different target features and criteria for alternative splicing detection, some of which represent distinct splicing patterns at different locations within the gene, which makes them impossible to relate in the absence of a reference set of gene and transcript annotations. To address this limitation, we used the gene as the unit of differential splicing information. Hence, for each program tested we consider the list of genes for which the program reports differentially spliced features, and compare it to the set of simulated genes.” (section “****Evaluation on simulated data****”).*

Q7. “Minor:
In the zero inflated NB, pi and mu are related to each other. If I am right, the chi-2 statistical test that compares log-likelihoods will be significant if any of the parameters of the NB changes (either mu, sigma or pi). Perhaps, events with low pvalues are not too interesting (for example a change in pi or in the variance). This consideration does not invalidate the analysis (in fact this is a minor comment) but shows the need to set a threshold in PSI.”

*Answer:*

Our likelihood-based formulation and the LLR test depend on all parameters, as well as the number of degrees of freedom. Therefore, it is indeed possible for parameters other than mu to cause a low p-value. Whether such a difference is of biological interest depends on the context. For instance, when comparing tumor versus normal samples it is not uncommon to observe differences where the mu values may not appear different, but the cancer group exhibits significantly higher variance due to biological variability. Such differences, when supported by medium or high read counts, are biologically significant. In other cases, for instance in comparisons of different cellular states where less biological heterogeneity is expected, or when testing a low expression gene where the estimates are made based on low read counts, a low p-value without the effect size (dPSI or log2FC) may not be informative. Imposing a minimum PSI (log2FC) cutoff could then help identify events with likely functional effects. We note that we applied a |dPSI|>=0.05 cutoff to deem an event significant for all MntJULiP applications described in the manuscript.

Q8. “One advantage of MntJULiP is the possibility to study several conditions at once. Is there any possibility to include also a continuous factor instead of an indicial one? Let me illustrate this with an example: would it be possible to find splicing events that are correlated with the body mass index for example? In other words, can a general design matrix be used with MntJULiP?”

*Answer:*

This could be a very interesting potential application, however there are limitations and significant effort involved, and we currently lack the resources and funding for such an undertaking. For example, one approach would be to bin the continuous factor’s values and convert to categorical terms, or ‘conditions’, and then directly use the multi-condition test at event level to identify differences in splicing. Note that the test would be able to reveal an association, but not a correlation, between different categories and splicing patterns, a limitation of our LLR test.

Q9. “For a more clear representation, the Venn diagrams could be proportional or using an Upset plot. There are some packages in R (and probably also in Python) easy to use, customizable and with a very good output for both upset and proportional Venn diagrams.”

*Answer:*

We thank the reviewer for pointing out these resources. However, since un-proportional diagrams are still the primary vehicles for conveying such information and describe the data fully, we decided not to change the displays at this time. Additionally, we also wanted to showcase our visualization suite Jutils, which has meanwhile been published, and which we have been using in generating our diagrams.

Q10. “There are wrong characters (question marks) in Figure 1C.”

*Answer:*

Thank you for pointing these out. We corrected these in the panel figure.

Q11. “Figure 1D. Perhaps the rows could be ordered using a tree to show more apparently the differences between the samples.”

*Answer:*

We changed **Figure 1D** to show the heatmap with rows clustered by the Ward method.


**REVIEWER #2:**
 
Q1.     “The metric for comparison between MntJULip and Cuffdiff2 is not quite clear as MntJulip is trying to quantify intron while cuffdiff2 quantifies isoform abundance.“

*Answer:*

Indeed, as we also mentioned in the manuscript, different tools use different metrics, objective functions and target features to define and determine differential splicing. Moreover, features identified by different tools may not be comparable at all - for example, when they do not co-locate, or when the class of events is not identifiable by one of the methods (see examples in **Supplementary Figure S5),** or when the actual gene and transcript set is not known**.** To overcome heterogeneity, we used the gene as the unit in our comparative evaluation. For more details, we kindly refer the reviewer to our answer to **Reviewer 1’**s question **Q6**.
 
Q2.     “As with most of the alternative splicing analysis algorithms, the program also consists of two parts, builder and quantifier.  The first step basically extracts all the introns while the second step conducts a statistical test on these introns. It would be revealing to see which step of the two contributes to the high sensitivity and high precision of the algorithm in comparison to other algorithms for both DSA and DSR detection.”

*Answer:*

This is a relevant and practical question. We note that the performance of programs depends on a multitude of factors, and that choices made in one section of the program may determine, or can be compensated by, choices in other sections. For instance, the builder may choose a more inclusive candidate set, whereas the quantifier may employ a more stringent test and additional filters, versus the builder may choose a more stringent set of features, coupled with a weaker test and/or set of filters in the quantifier. Therefore, the true contributions of each step may not be possible to fully deconvolve.

With this caveat, using the simulation experiment we measured the sensitivity and precision at each of the two steps, namely for the set of candidates produced by the builder (B) and the set of predictions produced by the quantifier (Q) (see the enclosed **Table** and **Figure**). As it can be seen, all programs but rMATS started with an almost complete set of the target genes in the candidate set, but the filters and tests within the quantifier reduced the sensitivity. Further, most programs started with a narrow set of candidates, whereas SUPPA2 and Cuffdiff2 started with a large and highly inclusive database. Sleuth and MntJULiP(DSA) showed the smallest change overall, and rMATS in the DSR category.

 
Q3.     In the simulated dataset, the authors claim that the high false-positive rates for MAJIQ, rMATs and Cuffdif2 were due to genes outside the simulated gene set. This in fact is a difficult problem. However, it is unclear from the method description how the MntJulip can address this specific challenge.

*Answer:*

MntJULiP addresses the problem of reducing false positives, including those from paralogs and spurious matches, in several ways that have been reported throughout the manuscript. First, with large numbers of samples (>10), introns with fewer than 3 reads are removed in each sample to reduce the sampling or alignment ‘noise’. Next, MntJULiP requires a minimum abundance of an intron across the samples in each condition, represented by the average read count, implemented in the parameter ‘min-count’. Third, a filter that removes introns with weak support within the gene’s context is embedded in the statistical model, by setting a cutoff value for the estimated read count. Lastly, inherent to *de novo* DSR methods is the fact that creating a group (or ‘bunch’) to test for differential splicing requires at least two introns that share a splice site, which significantly reduces the number of false positive from spurious matches, the effects of which can be seen for both MntJULiP and LeafCutter (n.b., the DSA component of MntJULiP produces considerably more false positives than its DSR one). To increase readability, we collected these explanations in one paragraph under ‘**Conclusions**’: *“Additionally, MntJULiP achieved improved accuracy, in particular precision, owing to its careful handling of low support introns, by filtering low abundance introns in individual samples and across all samples in a condition.”*
 
“It is also unclear why the transcript assembly from string-tie were fed to other algorithms for this experiment. String-tie itself might contain false-positive results thus affecting the accuracy of the algorithms relying on it as reference.”

*Answer:*

As noted in the manuscript, we used a modified transcript set to model the real-case scenario where the reference gene annotations provided as input to programs are inaccurate, namely: i) do not reflect the precise set of expressed transcripts, and ii) include a broad database consisting of all genes, which increases the potential for false positives. Most quantification programs perform well when the set of isoforms is known, but their performance drops for real data, where the expressed transcriptome in a cell type or tissue, or unique to the particular experiment, is unknown. Consistent with these observations, tools that made the least use of reference annotations, namely MntJULiP and LeafCutter, had the highest precision and best overall accuracy.
 
Q4.     “The authors claimed MntJUlip may detect a variety of alternative splicing events. But the algorithm is indeed intron centric. It only tells which intron is differentially expressed but really cannot tell what alternative splicing is going on. It will need a great deal of digging and interpretation to get a complete picture of alternative splicing events.”

*Answer:*

This is correct, and we kindly refer the reviewer to our answer to **Reviewer 1**’s question **Q6** where we addressed this question. Additionally, we re-iterate that there are important advantages to using an intron based approach, a strategy adopted by both LeafCutter and MntJULiP, including: i) not relying on an error-prone annotation (either a reference gene annotation that is not reflective of the data set and the expressed transcriptome, as explained in our answer to **Q3** above, or a set of transcript assemblies), ii) eliminating ambiguity in distinguishing among and quantifying disjoint splice patterns within a gene, and iii) ability to capture more splicing patterns than can be expressed through the known set of primitive alternative splicing events (exon skipping, alternative exon and gene ends, mutually exclusive introns).

Q5.     “There does not exist detailed description of different parameters used for other algorithms. It is not clear if the optimal setting for other algorithms has been attempted. For example, MntJULip used a threshold of 3 to filter introns. Did other algorithms also take similar threshold? Such threshold could be crucial for performance difference.“

*Answer:*

We used the default values for all methods, which are the settings chosen by the authors in their own experimentation as being optimal for most cases. (A complete list of commands used for all programs and all differential splicing analyses is now included in **Additional File 3**.) While it is indeed possible that some other settings may work better, evaluating and optimizing the parameter list for each application is a daunting task that is highly impractical, especially as some of the tools have extensive parameter lists, and is not generalizable.

For MntJULiP, most of the parameters (including the n>=3 cutoff) have already been tuned to work well for most data sets and are internal parameters only, which cannot be adjusted by the user. Importantly, (internal and default) parameter choices are judiciously decided by the methods’ developers based on tradeoffs to optimize the program performance. We are not aware of the equivalent parameters in other programs, and their choices. We refer the reviewer to our study above (see **Q2, Table** and **Figure**) for how the design choices within the ‘builder’ and ‘quantifier’ modulate the programs’ performance.
 
Q6.     “There are certain parameters required to be set within MntJUlip's statistical model. It is unclear how it was set in each experiment. It is interesting to see the performance as a function of different parameters including the minimum read count used to filter lowly expressed introns. “

*Answer:*

All internal parameters of the statistical model are ‘machine-learned’, using the Limited-memory Broyden–Fletcher–Goldfarb–Shanno (LM-BFGS) optimization method, for each application based on the input data. There is a very small number of other internal parameters, such as n>=3 filter for intron read count and beta>1.0 , which have been judiciously decided and whose values apply to all data sets. The main parameter that is user-accessible and can be varied is ‘min-count’, or the minimum cutoff for the average per condition read count for an intron. This value is set to 1 for the simulation experiment, where the data is less ‘noisy’ and shows less variability among replicates, and to 5 for analyses on sets of real data, which are more heterogeneous and have more inherent noise from spurious reads. For this reason, ‘min_count=5’ is the default parameter value for the program. We also note that varying the ‘min_count’ parameter for the simulation experiment (see **Table** below) changes the performance only slightly, with MntJULiP’s performance as measured by the F-value remaining above LeafCutter’s for ‘min_count’ values up to 20 for the DSR method, while MntJULiP(DSA)’s performance is comfortably above the others. Therefore, our program achieves robust high-performance results.

Q7.     “The figures reporting the performance comparison should only include algorithms in the same category, i.e., either DSA related algorithm or DSR related algorithm.”

*Answer:*

Our original intent with the combined figure was to illustrate that the two objective functions, DSR and DSA, model distinct classes of events, and that programs designed for one problem are not suitable to address the other. However, we agree that this may be confusing, and we already illustrate the difference between the two criteria with our gene functional analysis, therefore we replaced the figures in the revised manuscript as suggested.

**Second round of review**

**Reviewer 1**

The authors have addressed all my questions. Thanks for your work and congrats: it can be a very useful tool.
